# CAMSAP3 depletion induces lung cancer cell senescence‐associated phenotypes through extracellular signal‐regulated kinase inactivation

**DOI:** 10.1002/cam4.4380

**Published:** 2021-11-01

**Authors:** Onsurang Wattanathamsan, Paninee Chetprayoon, Naphat Chantaravisoot, Piriya Wongkongkathep, Pithi Chanvorachote, Varisa Pongrakhananon

**Affiliations:** ^1^ Inter‐Department Program of Pharmacology Graduate School Chulalongkorn University Bangkok Thailand; ^2^ Preclinical Toxicity and Efficacy Assessment of Medicines and Chemicals Research Unit Chulalongkorn University Bangkok Thailand; ^3^ Toxicology and Bio Evaluation Service Center National Science and Technology Development Agency Pathum Thani Thailand; ^4^ Department of Biochemistry Faculty of Medicine Chulalongkorn University Bangkok Thailand; ^5^ Center of Excellence in Systems Biology Faculty of Medicine Chulalongkorn University Bangkok Thailand; ^6^ Department of Pharmacology and Physiology Faculty of Pharmaceutical Sciences Chulalongkorn University Bangkok Thailand; ^7^ Cell‐based Drug and Health Product Development Research Unit Faculty of Pharmaceutical Sciences Chulalongkorn University Bangkok Thailand

**Keywords:** CAMSAP3, cellular senescence‐associated phenotypes, cyclin D1, extracellular signal‐regulated kinase 1/2 (ERK1/2), lung cancer

## Abstract

**Background:**

Cellular senescence is an aging‐related process found in cancer cells that contributes to irreversible growth arrest and tumor aggressiveness. Recently, calmodulin‐regulated spectrin‐associated protein 3 (CAMSAP3), a minus‐end microtubule‐stabilizing protein, has received increasing attention in cancer cell biology. However, the biological role of CAMSAP3 on senescence in human lung cancer remains incompletely understood.

**Methods:**

The function of CAMSAP3 on the regulation of cellular senescence‐associated phenotypes in human non‐small cell lung cancer H460 cells were determined in *CAMSAP3* deletion (H460/C3ko) cells. The effects of CAMSAP3 on cell proliferation were investigated using MTT and colony formation assays. The cell cycle activity was evaluated by flow cytometry and the senescence‐associated phenotypes were observed by SA‐β‐Gal staining. Quantitative RT‐PCR and westen blot were used to evaluate the expression of cell cycle and senescence markers. Moreover, the interaction of CAMSAP3‐ERK1/2 and possible partner protein was quantified using immunoprecipitation/mass spectrometry and immunofluorescence. Lastly, an xenograft model were performed.

**Results:**

*CAMSAP3* knockout promotes lung cancer cell senescence‐associated phenotypes and induces G1 cell cycle arrest. Mechanistic investigation revealed that phosphorylated ERK (p‐ERK) was markedly downregulated in *CAMSAP3*‐deleted cells, suppressing cyclin D1 expression levels, and full‐length CAMSAP3 abrogated these phenotypes. Proteomic analysis demonstrated that vimentin, an intermediate filament protein, is required as a scaffold for CAMSAP3‐modulating ERK signaling. Furthermore, an *in vivo* tumor xenograft experiment showed that tumor initiation is potentially delayed in *CAMSAP3* knockout tumors with the downregulation of p‐ERK and cyclin D1, resulting in a senescence‐like phenotype.

**Conclusion:**

This study is the first to report an intriguing role of CAMSAP3 in lung carcinoma cell senescence‐associated phenotypes via the modulation of p‐ERK/cyclin D1 signaling.

## Graphical abstract

Senescence is characterized by an irreversible growth arrest, and the biological role of senescence and its regulation in human lung cancer remains incompletely understood. This study demonstrated that *CAMSAP3* knockout promotes lung cancer cell senescence‐associated phenotypes through suppression of ERK/vimentin complex that is required for stabilization of ERK activity.

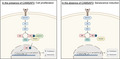

## 1 INTRODUCTION

Lung cancer is a common malignancy and the leading cause of cancer‐related deaths worldwide.^1^ The incidence and mortality rates are gradually increasing yearly, despite recent advances in therapeutic interventions. The unsatisfactory clinical outcomes are likely caused by multiple factors, including chemotherapeutic resistance, metastasis, and cancer relapse.^2^, ^3^ Identification of the molecular mechanisms regulating these aggressive cancer phenotypes is required to provide potential therapeutic targets for anticancer drug research and discovery.

Cellular senescence plays a dual role in cancer pathogenesis and has both tumor‐suppressive and ‐promoting effects.^4^, ^5^ Although cellular senescence, an irreversible cell cycle arrest linked to the aging process, is widely accepted as a tumor‐suppressive mechanism, recent studies have reported that cellular senescence‐like phenotypes in cancer cells contribute to cancer progression via the development of the senescence‐associated secretory phenotype (SASP).^6^, ^7^, ^8^ The dual role of cellular senescence‐like phenotypes in cancer is considered a double‐edged sword, and its exact function and mechanism, particularly in lung cancer, are not yet completely understood. Several signaling pathways have been shown to control cellular senescence in association with oxidative stress, oncogenes, DNA injury, and telomerase dysfunction.^9^ Extracellular signal‐regulated protein kinase (ERK) plays an essential role as a messenger in response to both intracellular and extracellular stimuli.^10^ ERK is a regulator of cell proliferation; however, recently, it has been reported as a crucial mediator of senescence.^10^ Accumulating studies have revealed that ERK activation can enhance cellular senescence‐like phenotypes by inducing the expression of the tumor suppressor protein p21.^11^ By contrast, ERK suppression promotes a senescence‐like phenotype in human cancer cells by several mechanisms.^12^, ^13^, ^14^ Nonetheless, the regulatory effect of ERK signaling on senescence is cell‐type specific and depends on the coordinated pathway and dynamics of active ERK.^15^ The identification of the molecules involved in this process might provide a better understanding of ERK regulation and the cancer biology of senescence.

Recently, the calmodulin‐regulated spectrin‐associated protein (CAMSAP) family has gained increasing attention in cancer cell biology.^16^ CAMSAPs, non‐centrosomal microtubule minus‐end binding proteins, are required for maintaining microtubule organization and cellular morphogenesis.^16^, ^17^, ^18^ The CAMSAP family comprises CAMSAP1, CAMSAP2, and CAMSAP3, and we reported that CAMSAP3 maintains the epithelial state of lung cancer‐derived cells, preventing them from epithelial–mesenchymal transition (EMT), which is a form of cell changes associated with cancer progression.^19^ Gene expression profiling data have shown that lung cancer tissues with low CAMSAP3 expression levels are closely associated with a decreasing overall survival rate of lung adenocarcinoma patients.^20^ Furthermore, CAMSAP3 levels decline in advanced‐stage cancer, suggesting that CAMSAP3 functions as an antimetastatic regulatory protein.^20^ However, the biological role of CAMSAP3 in lung cancer remains largely unknown. A previous study reported that *CAMSAP3* knockout cells undergo cell growth arrest, depending on cell culture conditions,^19^ and cellular senescence is a key aggressive phenotype related to cell proliferation; however, no evidence is available demonstrating a relationship between CAMSAP3 and cancer senescence. This study aimed to investigate the biological role of CAMSAP3 in cellular senescence and explore the underlying mechanism, particularly the involvement of the ERK signaling pathway in NSCLC cells in vitro and xenograft‐immunodeficient mice in vivo.

## 2 MATERIALS AND METHODS

### 2.1 Cells and reagents

NSCLC NCI‐H460 and NCI‐A549 cells were purchased from American Type Culture Collection (ATCC). *CAMSAP3* knockout H460 (H460/C3ko) and control (H460/Ctrl) cells were generated as previously reported.^19^ The antibody against CAMSAP3 was a gift from Professor Takeihi Masatoshi (RIKEN) and others were provided in Table S2.

### 2.2 Proliferation and cell growth assay

A 5 × 10^3^ cells/well were seeded into 96‐well plates for 24 and 48 h. The MTT solution was added to each well and the absorbance was examined after incubating at 37℃ for 4 h using a microplate reader (Perkin Elmer VICTOR3/Wallac 1420). The proliferation was calculated relatively to those of the initial time. For cell growth assays, 3 × 10^3^ cells/well were cultured onto 24‐well plates. The cells were trypsinized and counted for 7 days continuously using the LUNA II automated cell counter (Logos Biosystems). The doubling time was calculated as previously described.^21^

### 2.3 Cell cycle analysis

After 1 × 10^5^ cells were fixed in cold methanol at 4°C overnight, they were incubated with 100 µg/ml of RNase A and 50 µg/ml of propidium iodide at room temperature for 30 min. The DNA content was evaluated by flow cytometry using an EPICS‐XL flow cytometer (Beckman Coulter), and the number of cells in each phase of the cell cycle was plotted. All the data analyses were performed using at least 20,000 gated events/samples.

### 2.4 Clonogenic assay

A total of 5 × 10^3^ cells was cultured onto a six‐well plate for 14 days, replacing the medium with fresh medium every 2 days. The cells were stained with 0.01% crystal violet in 10% ethanol at room temperature for 30 min as previously described.^22^ To evaluate the number of cells, after washing five times with deionized water, the cells were incubated using the nuclear staining dye DAPI, counted, and represented as an average number of cells per colony.

### 2.5 SA‐β‐gal staining assay

The cells were fixed with a fixative solution at room temperature for 15 min. After that, the cells were washed twice in PBS for 5 min each and incubated in a senescence‐associated β‐galactosidase (SA‐β‐gal) staining solution for 24 h at 37ºC in the absence of CO_2_. SA‐β‐gal staining was microscopically observed as blue‐stained positive cells. The SA‐β‐gal‐positive cells were counted from at least five random fields with at least 500 cells/group, and the data were presented as a percentage of positive SA‐β‐gal cells to the total cell number.

### 2.6 Biochemical assays

Methods for plasmid transfection, RNA extraction and qRT‐PCR, western blot analysis, immunofluorescence, immunoprecipitation, and mass spectrometry and immunohistochemistry are described in Supplementary Methods. The primers for qRT‐PCR are shown in Table S1 and antibodies used are listed in Table S2.

### 2.7 Tumor xenograft studies

Six‐week‐old male BALB/cMlac‐nu nude mice were purchased from Nomura Siam International (Bangkok, Thailand). The study was approved by the Institutional Animal Care and Use Committee of the Blind for review, (CU‐AUP 19–33–003). Both flanks of a mouse were subcutaneously inoculated with 5 × 10^6^ cells of either H460/Ctrl or H460/C3ko cells. The mice were weighed, and the tumor volume was calculated as follows: (length × width × width)/2. After 45 days, the mice were euthanized and the tumors were dissected, fixed with 4% paraformaldehyde for 24 h for histologic observation, and stored at −20°C for further biochemical assays.

### 2.8 Statistical analysis

All the data were presented as mean ± SEM obtained from at least three independent experiments. Statistical analysis was performed using unpaired Student's *t*‐test or the Mann–Whitney *U*‐test using Prism 8 (GraphPad software). *p*‐value < 0.05 were considered statistically significant.

## 3 RESULTS

### 3.1 *CAMSAP3* deletion inhibits the growth of NSCLC H460 cells

**FIGURE 1:**
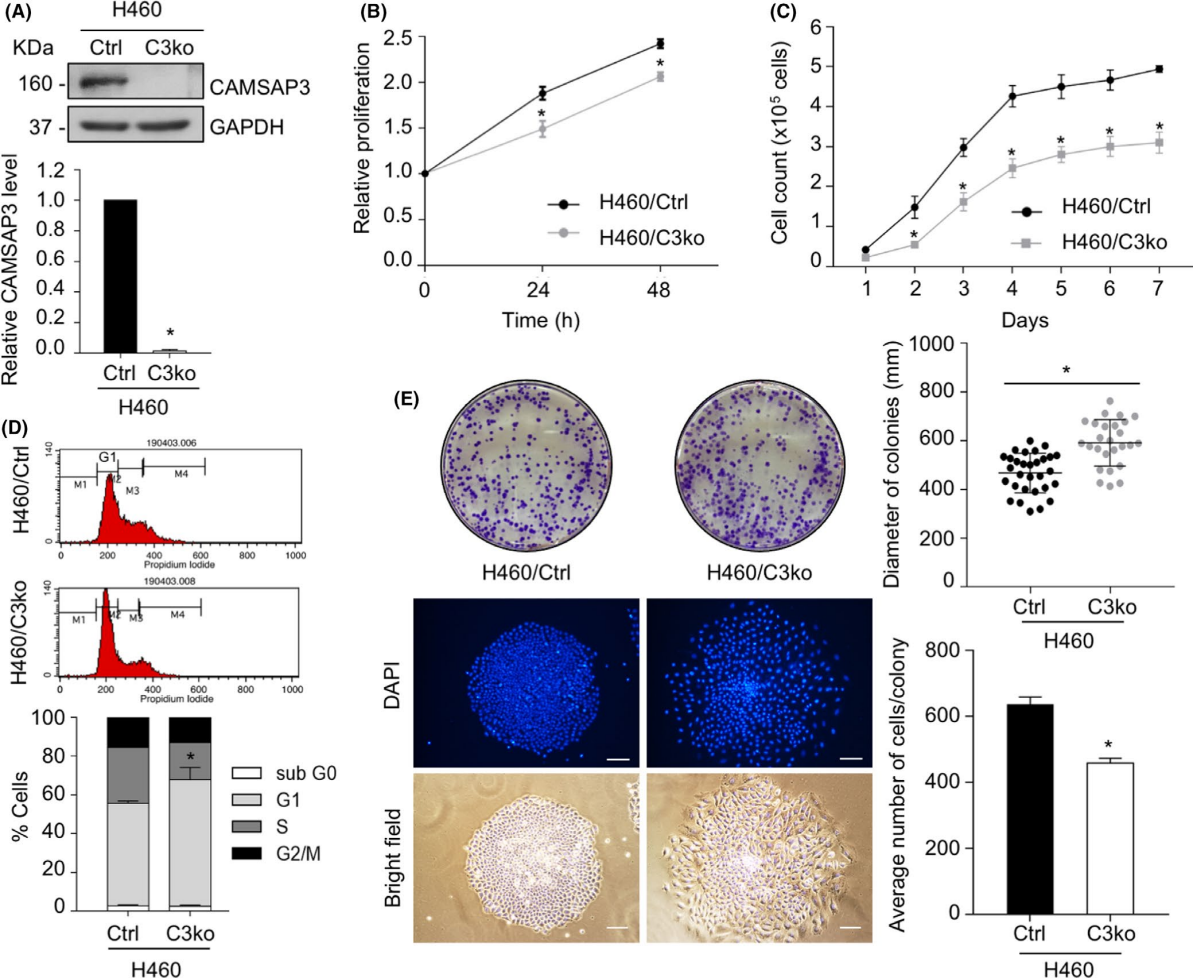
*CAMSAP3* depletion inhibits cell growth in NSCLC. (A) Western blotting for CAMSAP3 expression in H460 control (H460/Ctrl) cells and *CAMSAP3* knockout H460 (H460/C3ko) cells. (B) Cell proliferation of H460/Ctrl and H460/C3ko cells was examined by the MTT assay at 0, 24, and 48 h. (C) Cell growth of H460/Ctrl and H460/C3ko cells was evaluated by counting at each time point. (D) The percentage of cells in each phase of the cell cycle was analyzed by flow cytometry. (E) Cell growth was determined by the clonogenic assay. The colony diameter was measured using ImageJ software and presented as the mean colony diameter ± SEM. The number of cells/colonies was evaluated by DAPI nuclear staining and presented as the average number ± SEM. Student's *t*‐test, (*n *= 30 for each H460/Ctrl and H460/C3ko cells). Scale bars: 10 µm. Data were represented as mean ± SEM. *p*‐values were calculated using a Student's *t*‐test *p < 0.05 vs. H460/Ctrl cells

We previously isolated *CAMSAP3* knockout (H460/C3ko) cells using the CRISPR‐Cas9 system (Figure 1A) and found that, in the absence of CAMSAP3, cell growth was attenuated when they were cultured as monolayers, but it was exerted on soft agar.^19^ Because anchorage‐independent growth is an essential feature of mesenchymal‐like phenotypes,^23^ to minimize the interference of such behavior on cellular senescence‐associated phenotype, we performed cell proliferation and cell growth assays in NSCLC cells in an attachment culture. H460/C3ko cells had a slower cell proliferation rate with a 43.44‐h doubling time than control (H460/Ctrl) cells, which had a 23.17‐h doubling time (Figure 1B, C). We next examined whether CAMSAP3 deletion causes changes in the proportion of cells in various phases of the cell cycle in NSCLC. Cell cycle analysis demonstrated that the loss of CAMSAP3 specifically induces cell cycle arrest at the G1 phase, as evidenced by an increase in up to 22.3% of H460/C3ko cells (Figure 1D). To confirm cell growth arrest in H460/C3ko cells, long‐term cell growth was evaluated using a clonogenic assay. Although the average diameter of the H460/C3ko colonies increased up to 1.2‐fold compared with that of H460/Ctrl cells, the average number of cells per colony was significantly decreased in H460/C3ko cells (Figure 1E). These results suggest that the loss of CAMSAP3 suppresses cell growth, inducing cell cycle arrest at the G1 phase.

### 3.2 *CAMSAP3* loss induces senescence‐associated phenotypes

**FIGURE 2:**
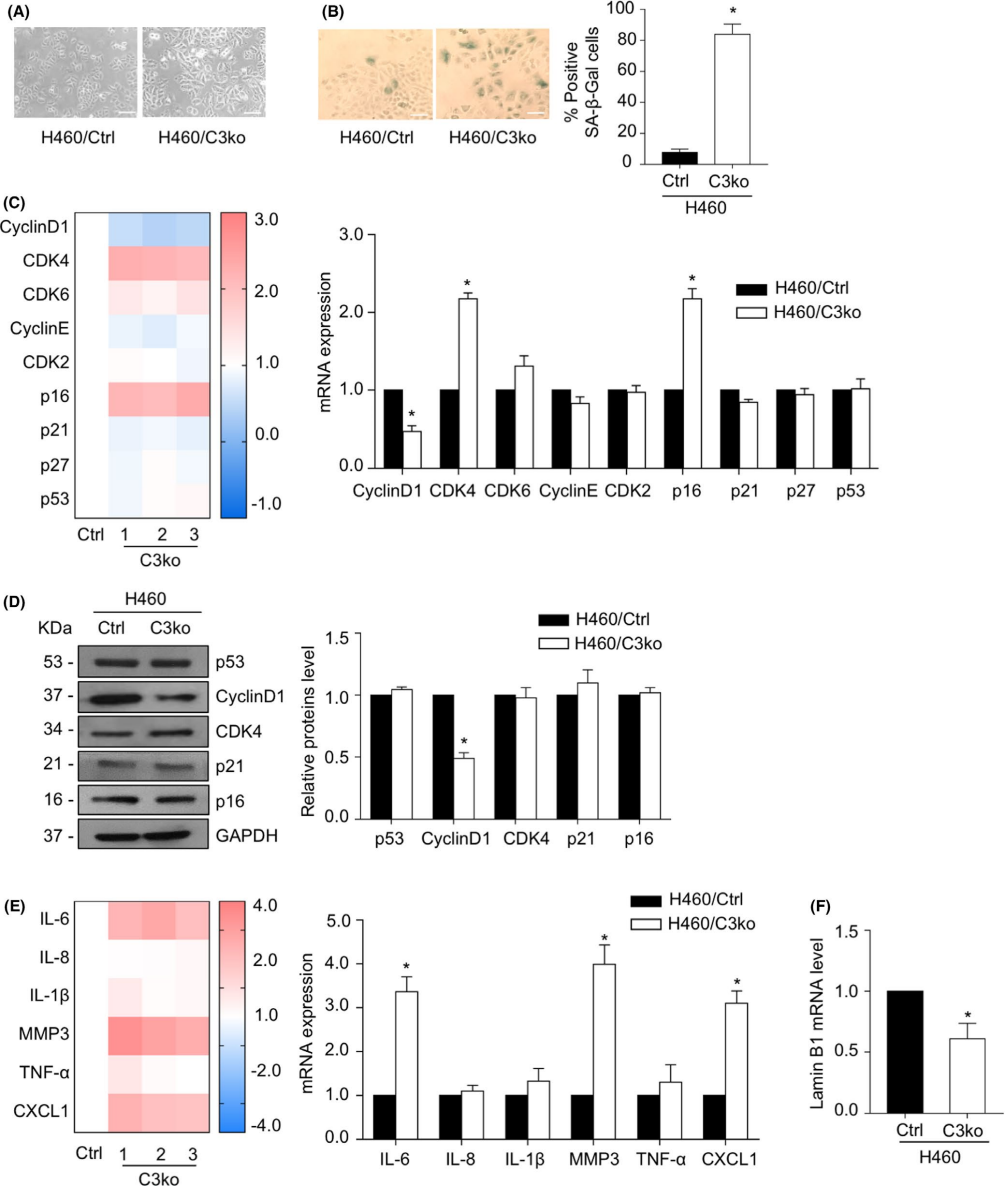
*CAMSAP3* loss mediates lung cancer cell senescence‐associated phenotypes. (A) Phase‐contrast images of H460/Ctrl and H460/C3ko cells. Scale bars: 10 µm. (B) Representative phase‐contrast images of SA‐β‐gal‐stained cells (positive blue‐stained cells) and percentage of positive SA‐β‐gal cells. Scale bars: 10 µm. (C) The mRNA expression levels of cell cycle and senescence regulator genes were measured by quantitative RT‐PCR. The heatmap represented the mRNA levels relative to those of H460/Ctrl cells. (D) Western blot for cell cycle and senescence regulator proteins in H460/Ctrl cells and H460/C3ko cells. The intensity was normalized to that of GAPDH. (E) The mRNA expression levels of SASP were measured by quantitative RT‐PCR. The heatmap represented the mRNA levels relative to those of the H460/Ctrl cells. (F) The mRNA expression level of lamin B1 was measured by quantitative RT‐PCR. Data were presented as mean ± SEM. *p*‐values were calculated using a Student's *t*‐test *p < 0.05 vs. H460/Ctrl cells

Because flattened cell bodies and enlarged cell sizes are essential characteristics of senescent cells,^5^ we then re‐examined the cellular morphology of *CAMSAP3*‐deleted cells. Notably, *CAMSAP3* knockout cells appeared slightly larger, with an average cell diameter of 16.02 ± 1.30 µm, whereas H460/Ctrl cells had an average cell diameter of 13.08 ± 0.62 µm (Figure 2A). Next, we assessed the senescence‐associated (SA)‐β‐galactosidase activity, a marker of senescent cells. *CAMSAP3* deletion induced SA‐β‐gal activity, whereas the number of SA‐β‐gal‐stained cells increased to approximately 84% compared with 7.67% of H460 control cells (Figure 2B). The mRNA and protein levels of key senescence regulatory factors were evaluated. The mRNA expression level of cyclin D1 was significantly decreased, whereas the CDK4 and p16 mRNA expression levels were elevated in *CAMSAP3*‐deleted cells (Figure 2C). However, western blot analysis demonstrated that only cyclin D1, an important regulatory protein in the G1 phase of the cell cycle, was notably downregulated in H460/C3ko cells, whereas the expression levels of the other factors were unchanged (Figure 2D). Additionally, SASP expression, particularly IL‐6, MMP3, and CXCL1 expression, was extensively upregulated in the absence of CAMSAP3 (Figure 2E), and a decreased lamin B1 expression as a result of nuclear envelope integrity disruption, a senescence‐associated phenotype, was clearly observed in *CAMSAP3*‐deleted cells comparing to the control cells (Figure 2F).

We investigated cell growth arrest and cellular senescence‐associated phenotypes using RNA interference to specifically silence CAMSAP3 (siC3) in H460 and A549 cells, another lung carcinoma cell line. Western blot analysis confirmed the downregulation of CAMSAP3 levels after siRNA transfection (Figure S1A, B). Additionally, CAMSAP3 knockdown in both cell lines resulted in slower cell proliferation (Figure S1C, D) and upregulated senescence‐like phenotypes (Figure S1E, F). Furthermore, cyclin D1 expression was strongly downregulated in both CAMSAP3 knockdown H460 and A549 cells (Figure S1A, B). These findings suggested that CAMSAP3 knockdown induced cellular senescence‐like phenotypes, at least in part, through cyclin D1 downregulation in NSCLC cells.

To confirm the above finding, we stably expressed His‐tagged wild‐type CAMSAP3 in H460/C3ko cells (defined as C3ko/C3‐His cells) and mock control (C3ko/Ctrl) cells and investigated whether it could reverse the senescence‐like phenotype conferred by *CAMSAP3* deletion. Western blot assays confirmed the exogenous expression of CAMSAP3‐His in C3ko/C3‐His cells (Figure S2A). Morphologically, C3ko/C3‐His cells exhibited clusters of cells with strong cell–cell interactions (Figure S2B). CAMSAP3 overexpression was also reverse cell growth suppression (Figure S2C) and attenuated senescence‐associated phenotypes (Figure S2D). The number of SA‐β‐gal‐positive cells was markedly decreased in the C3ko/C3‐His group to approximately 12.3% compared with 78.3% in the C3ko/Ctrl group (Figure S2D). Conversely, the levels of cyclin D1 mRNA and protein were increased after introducing exogenous CAMSAP3 into *CAMSAP3* knockout cells (Figure S2E, F) but other cell cycle regulatory proteins were not significantly altered. Furthermore, the upregulation of SASP expression mediated by *CAMSAP3* loss, such as the upregulation of IL‐6, MMP3, and CXCL1 in C3ko/Ctrl cells, was significantly reduced because of CAMSAP3 overexpression (Figure S2G). These results confirm the hypothesis that CAMSAP3 negatively regulates cellular senescence‐associated phenotypes in lung carcinoma cells.

### 3.3 *CAMSAP3* knockout attenuates ERK activity

**FIGURE 3:**
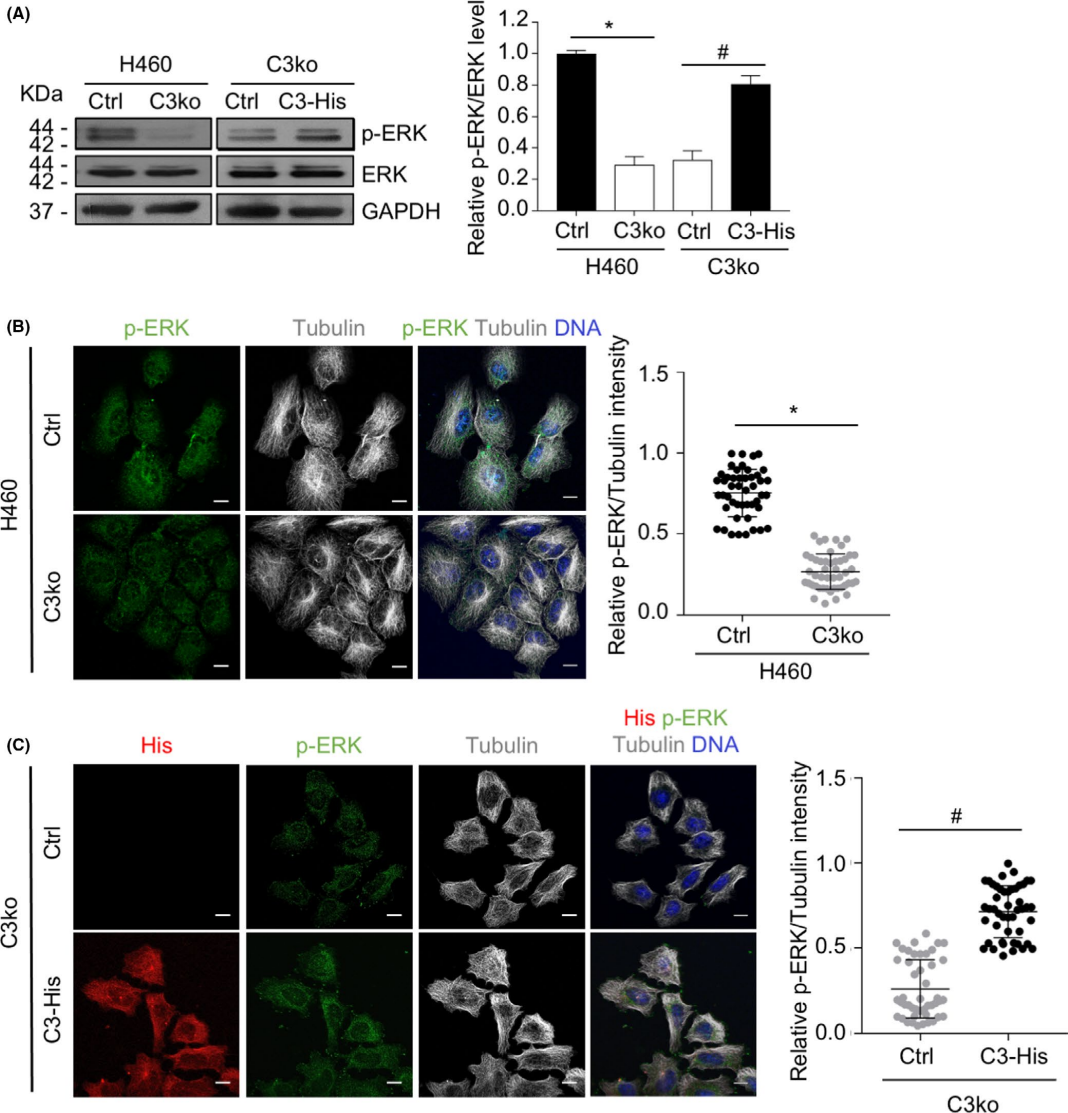
CAMSAP3 negatively regulates ERK activity. (A) Western blotting for p‐ERK and ERK in H460/Ctrl, H460/C3ko, C3ko/Ctrl, and C3ko/C3‐His cells. (B) Immunostaining for p‐ERK (green), α‐tubulin (grey), and DNA (DAPI, blue) in H460/Ctrl and H460/C3ko cells. The graph depicts the relative p‐ERK level to that of tubulin from each individual cell (*n *= 30). Scale bars: 10 µm. (C) Immunostaining for His‐tagged CAMSAP3 (His, red), p‐ERK (green), α‐tubulin (gray), and DNA (DAPI, blue) in C3ko/Ctrl and C3ko/C3‐His cells. The graph depicts the relative p‐ERK level to that of tubulin from each individual cell (*n *= 30). Scale bars: 10 µm. Data were presented as mean ± SEM. *p*‐values were calculated using a Student's *t*‐test *p < 0.05 vs. H460/Ctrl cells: ^#^ *p* < 0.05 vs. C3ko/Ctrl cells

ERK signaling plays a crucial role in cell proliferative‐related activity including senescence and regulates the transcription of cyclin D1.^11^ Therefore, we hypothesized that CAMSAP3 might alter ERK activation. Then, we investigated the association between ERK and CAMSAP3. Western blot analysis revealed that ERK phosphorylation was substantially downregulated in H460/C3ko cells but was restored after introducing exogenous wild‐type CAMSAP3 into *CAMSAP3* knockout cells; however, the total ERK level was not changed in the tested cells (Figure 3A). Consistent with the results in A549 and H460 cells with CAMSAP3 knockdown, the decrease in ERK phosphorylation was also clearly detectable (Figure S1A, B), suggesting that CAMSAP3 normally functions to regulate ERK activity. The immunostaining assay confirmed that phosphorylated ERK (p‐ERK), which exhibited punctate signals in H460 control (H460/Ctrl) cells, was greatly reduced after *CAMSAP3* knockout (Figure 3B). Furthermore, exogenous His‐tagged CAMSAP3 could rescue the p‐ERK signal in *CAMSAP3*‐deleted cells (Figure 3C).

We further investigated whether the upstream MAP kinase signaling was affected by *CAMSAP3* loss. The result demonstrated that ERK phosphorylation was upregulated in response to epidermal growth factor (EGF) in H460/Ctrl cells, but it was not altered in *CAMSAP3* depletion cells. Whereas EGF strongly activated MEK1/2 in both cells, indicating that CAMSAP3 regulates ERK activity specifically, without an effect on an upstream MAP kinase pathway (Figure S3).

**FIGURE 4:**
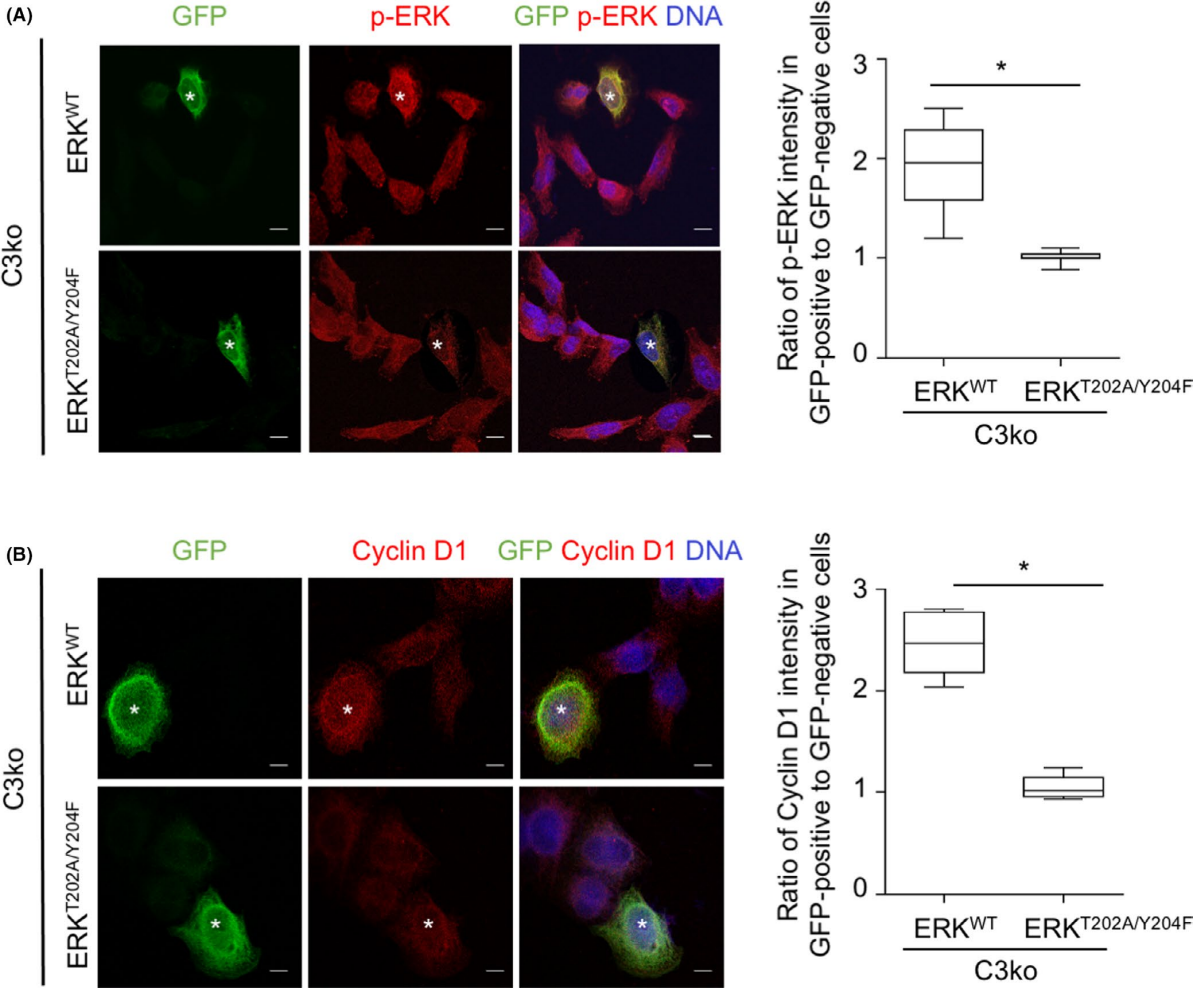
ERK overexpression increases cyclin D1 expression in *CAMSAP3*‐knockout cells. (A) H460/C3ko cells were transfected with either GFP‐ERK wild‐type (ERK^WT^) or GFP‐ERK dominant‐negative mutant (ERK^T202A/Y204F^) plasmid, and immunostained for GFP (green), p‐ERK (red), and DNA (DAPI, blue). The box plot shows the ratio of the *p*‐ERK intensity in GFP‐positive to ‐negative cells in each group. The asterisk indicates GFP‐positive cells (*n *= 20 cells). (B) H460/C3ko cells were transfected with either GFP‐ERK wild‐type (ERK^WT^) or GFP‐ERK dominant‐negative mutant (ERK^T202A/Y204F^) plasmid and immunostained for GFP (green), cyclin D1 (red), and DNA (DAPI, blue). The box plot shows the ratio of the cyclin D1 intensity in GFP‐positive to ‐negative cells. The asterisk indicates GFP‐positive cells (*n *= 20 cells). Scale bars: 10 µm. Data were presented as mean ± SEM. *p*‐values were calculated using a Student's *t*‐test *p < 0.05 vs. C3ko/ ERK^WT^ cells

To confirm this finding, H460/C3ko cells were transiently transfected with either GFP‐tagged wild‐type ERK plasmid (ERK^WT^) or dominant‐negative ERK mutant plasmid (ERK^T202A/Y204F^), in which the phosphorylation sites were disrupted by site‐directed mutagenesis. Because of the low transfection efficiency, we studied individual cells by immunostaining instead of using western blotting of whole‐cell lysates. p‐ERK increased in GFP‐positive ERK^WT^‐overexpressing *CAMSAP3* knockout (C3ko/ERK^WT^) cells compared with that in GFP‐negative cells, whereas no significant alteration of p‐ERK was observed in GFP‐positive and ‐negative ERK^T202A/Y204F^ transfectants (C3ko/ERK^T202A/Y204F^ cells) (Figure 4A). Furthermore, immunostaining revealed that cyclin D1 staining was clearly increased in GFP‐positive C3ko/ERK^WT^ cells, whereas its signal was not different between the GFP‐positive and GFP‐negative C3ko/ERK^T202A/Y204F^‐transfected groups (Figure 4B). Collectively, these observations suggest that *CAMSAP3* depletion may have induced cellular senescence‐associated phenotypes by suppressing ERK activity.

### 3.4 CAMSAP3 regulates ERK activity by promoting the vimentin‐p‐ERK complex

**FIGURE 5:**
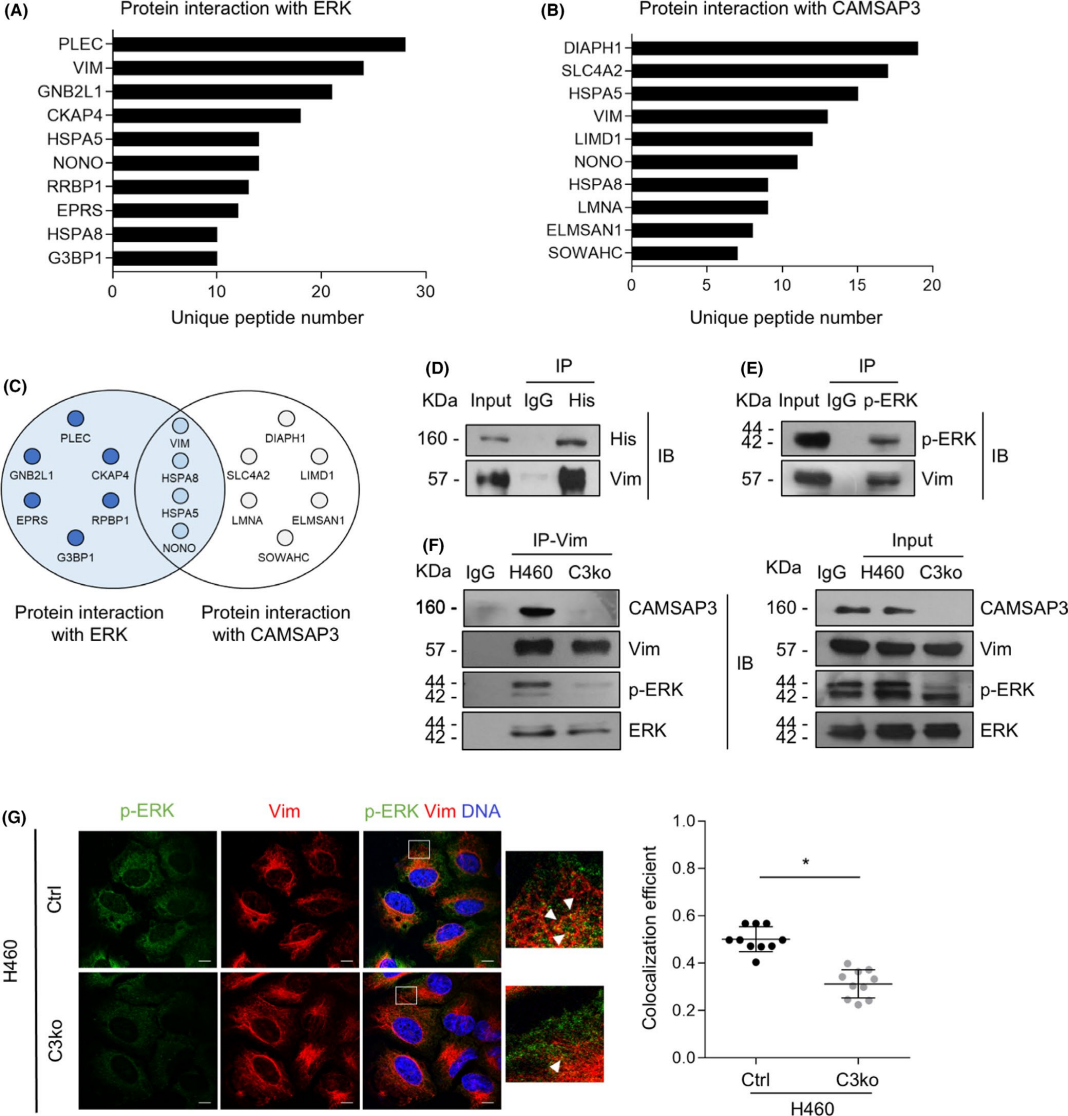
CAMSAP3 regulates ERK activity via vimentin. The top ten lists of proteins possibly interacting with (A) ERK and (B) CAMSAP3 were examined by immunoprecipitation and mass spectrometry (IP‐MS). The top ten possible partner proteins with the number of unique peptides more than three were listed. (C) Venn diagram of the overlapping peptides identified from IP‐MS analysis of ERK and CAMSAP3. (D) His‐tagged CAMSAP3 and (E) p‐ERK were pulled down by specific antibodies or IgG as a negative control, and then immunoblotted for vimentin. (F) Protein lysates prepared from H460/Ctrl to H460/C3ko cells were used for immunoprecipitation with antibody to vimentin or IgG as a negative control and then immunoblotted for CAMSAP3, vimentin, p‐ERK, and ERK. In the input blot, cell lysates were immunoblotted with antibodies against CAMSAP3, vimentin, p‐ERK, and ERK (right panel). (G) Immunostaining for p‐ERK (green), vimentin (red), α‐tubulin (grey), and DNA (DAPI, blue) in H460/Ctrl and H460/C3ko cells. The colocalization efficiency between p‐ERK and vimentin was analyzed using Manders’ coefficient, and the data were represented as mean ± SEM. Student's *t*‐test, *p < 0.05 (*n *= 20 cells). Scale bars: 10 µm

Next, we tested the hypothesis that CAMSAP3 may directly interact with ERK and prevent its activation. We introduced His‐tagged CAMSAP3 plasmids into H460 cells (designated H460/C3‐His cells). His‐tagged CAMSAP3 was immunoprecipitated and immunoblotted for both p‐ERK and total ERK. No coprecipitation of CAMSAP3 was observed with either ERK or p‐ERK (Figure S4A). Additionally, an immunostaining assay confirmed the absence of colocalization between them (Figure S4B), indicating that CAMSAP3 regulates ERK activity without direct binding. To gain insight into the molecular mechanism by which CAMSAP3 inhibits ERK activity, we performed proteomics analysis to identify the potential interacting partners of these two proteins. Immunoprecipitation and mass spectrometry analysis (IP‐MS) were performed by pulling down proteins that interact with either His‐tagged CAMSAP3 or ERK and identifying them by mass spectrometry. A minimum of three unique peptides per positive protein identification was set as the criterion. One hundred ninety‐six proteins were found to possibly interact with CAMSAP3 and 193 proteins with ERK. The top ten peptides of each group were plotted (Figure 5A, B). Finally, 95 partner peptides were found in both groups, and the most likely partners are represented in a Venn diagram (Figure 5C). The results indicated that vimentin, an intermediate filament protein, potentially interacted with CAMSAP3 and ERK. Furthermore, immunoprecipitation assays demonstrated that vimentin clearly coprecipitated with both His‐tagged CAMSAP3 and p‐ERK (Figure 5D, E).

Because vimentin enhances ERK activation by binding to ERK and preventing ERK dephosphorylation by the alkaline phosphatase enzyme,^24^ we hypothesized that CAMSAP3 enhanced ERK activity by maintaining the vimentin‐p‐ERK interaction. Vimentin was then pulled down and immunoblotted with an antibody against p‐ERK in H460/Ctrl and H460/C3ko cells. The p‐ERK‐vimentin complex found in H460/Ctrl cells was obviously decreased in H460/C3ko cells (Figure 5F). Furthermore, immunofluorescence assays showed that the colocalization of p‐ERK puncta with vimentin was reduced significantly (Figure 5G), indicating that CAMSAP3 regulated ERK activation by promoting the vimentin‐p‐ERK interaction.

### 3.5 *CAMSAP3* depletion induces senescence‐associated phenotypes in an in vivo tumor xenograft

**FIGURE 6:**
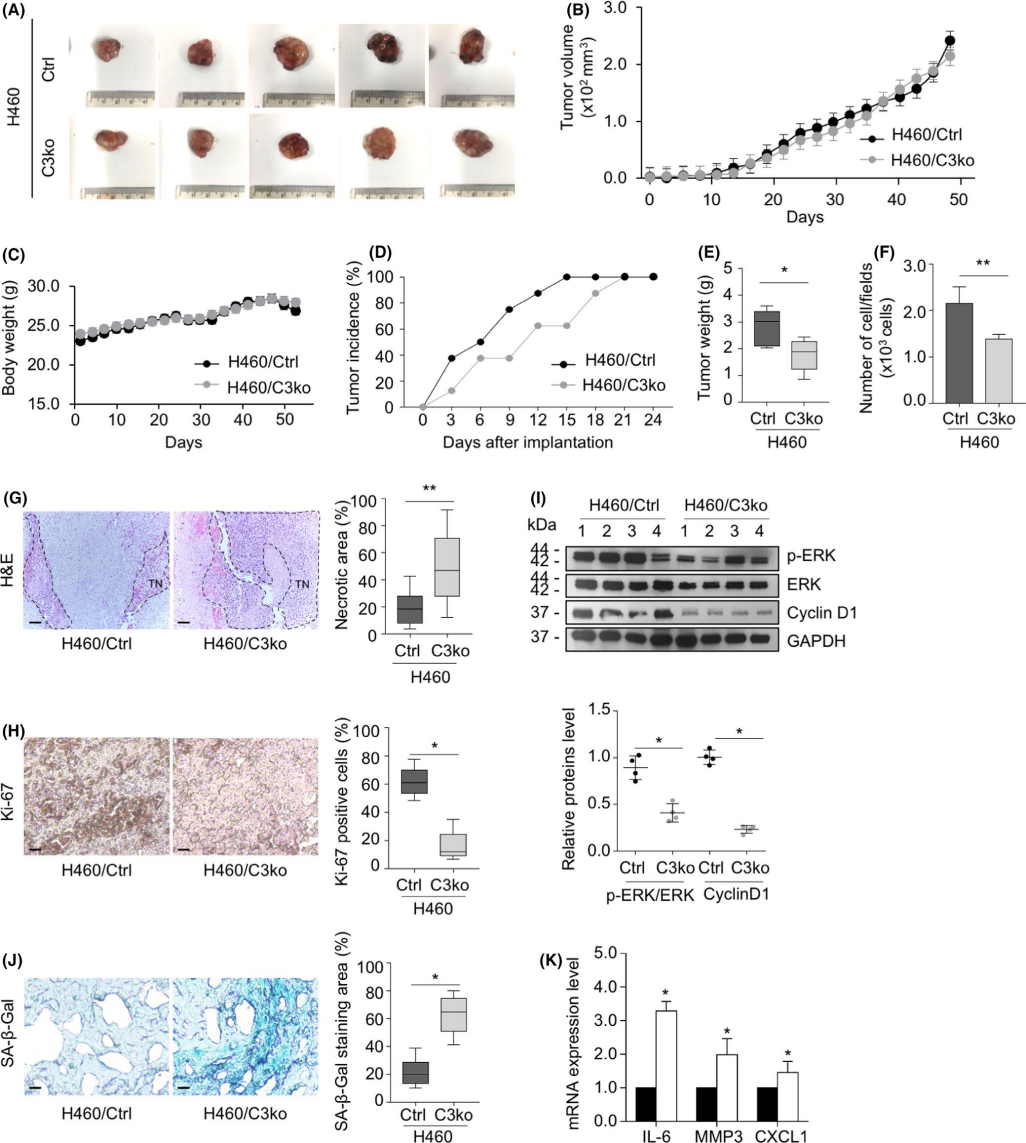
*CAMSAP3* depletion mediates senescence‐associated phenotypes in vivo in xenograft‐immunodeficient mice. (A) H460/Ctrl and H460/C3ko cells were subcutaneously injected into both flanks of 6‐week‐old nude mice. After 45‐day inoculation, H460/Ctrl (upper row) and H460/C3ko (lower row) xenograft tumors were dissected and photographed. (B) Graph of the average xenograft tumor volume of the H460/Ctrl and H460/C3ko groups (*n *= 10). (C) Graph of mouse body weights (*n *= 10). (D) The percentage of the tumor incidence in H460/Ctrl and H460/C3ko groups was plotted (*n *= 10). (E) The box plot data of tumor weights (g) and (F) The number of cells per field from H460/Ctrl and H460/C3ko xenograft tissues (*n *= 10). (G) Representative images of hematoxylin & eosin (H&E) staining were examined by bright field microscopy; TN indicates tumor necrosis and the percentage of the tumor necrotic area of the total tumor area from H460/Ctrl and H460/C3ko xenograft tissue (*n *= 10). Scale bars: 100 µm. (H) Representative images of immunohistochemistry staining for ki67 and the percentage of ki‐67‐positive cells to the total cell number from H460/Ctrl and H460/C3ko xenograft tissue (*n *= 10). Scale bars: 100 µm. (I) Tumor xenografts were collected and subjected to Western blot analysis for p‐ERK, ERK, cyclin D1, and GAPDH. The intensity was normalized to that of GAPDH (*n *= 4). (J) Immunohistology of SA‐β‐gal activity in H460/Ctrl and H460/C3ko tumor sections derived from xenografted mice. The intensity of SA‐β‐gal staining subtracted by the background was quantified, calculated as a percentage of the SA‐β‐gal‐positive area (*n *= 10). Scale bars: 100 µm. (K) The mRNA expression levels of IL‐6, MMP3, and CXCL1 were measured by quantitative RT‐PCR. The plot represents the relative mRNA level compared with that of the H460/Ctrl group (*n *= 4). Data were presented as mean ± SEM. *p*‐values were calculated using a Student's *t*‐test *p < 0.05 vs. H460/Ctrl cells: **p < 0.001 vs. H460/Ctrl group

We next investigated whether the loss of CAMSAP3‐induced senescence‐associated phenotypes *in vivo* in xenograft‐immunodeficient mice. After the subcutaneous injection of either H460/Ctrl or H460/C3ko cells into both flanks of each mouse, H460/Ctrl and H460/C3ko tumor nodules grew to a similar size on day 45 after implantation (Figure 6A, B), and the mouse weights were not altered (Figure 6C). When examined on day 3 after implantation, tumor nodules were observed in 37.5% of the H460/Ctrl group, and their formation was completed by day 15 (Figure 6D). However, the H460/C3ko group exhibited a longer latency time‐that is, tumors were detectable only in 12.5% of the mice on day 3 and fully developed on day 21 (Figure 6D), indicating that CAMSAP3 may play a role in tumor initiation. Interestingly, the tumor weight of the H460/C3ko group was significantly lower than that of the control (Figure 6E). Because the tumor cells in nodules comprise both proliferating and dead cells, H&E staining was then performed to investigate tissue morphogenesis. Interestingly, the number of cells per field was lower in the *CAMSAP3*‐deleted group than in the control group (Figure 6F), and the tumor necrotic area observed in the H460/C3ko group appeared to increase approximately up to 2‐fold compared with that in the H460/Ctrl group (Figure 6G). The expression levels of the proliferative marker ki67 were decreased in the H460/C3ko group (Figure 6H). Similarly, the levels of p‐ERK/ERK and cyclin D1 were markedly downregulated in H460/C3ko xenograft tumors compared with those in the H460/Ctrl group (Figure 6I). Additionally, SA‐β‐gal staining and SASP expression were increased in H460/C3ko xenograft tissue (Figure 6J, K). Furthermore, to investigate the correlation of the necrosis‐ki67 and necrosis‐SA‐β‐gal level in the H460/C3ko group, Pearson's correlation analysis was performed. The necrosis level was negatively correlated with ki67 expression at the correlation coefficient of 0.8373 (Figure S5A), whereas SA‐β‐gal showed a positive relationship with the necrosis area with a correlation coefficient of 0.9752 (Figure S5B). These data suggest that prolonged xenograft generation contributes to necrotic cell death in the *CAMSAP3* knockout group consistent with a lower ki67 level and higher SA‐β‐gal staining, indicating the regulatory effect of CAMSAP3 on senescence‐associated phenotypes. Taken together, these results indicated that *CAMSAP3* removal induces senescence‐associated phenotypes in an in vivo xenograft, supporting our finding in the in vitro study showing that CAMSAP3 negatively regulates cellular senescence‐associated phenotypes via ERK/cyclin D1 signaling.

## 4 DISCUSSION

Cellular senescence is a cellular process of irreversible growth arrest that occurs when cells undergo oncogenic stress.^5^ Because senescence can restrict the propagation of damaged cells, a possible risk factor for tumor transformation, it is considered a tumor suppressor mechanism.^6^, ^7^ Nevertheless, this function is not the only characteristic of senescence activity. Regardless of their proliferative arrest, senescent cells remain metabolically active and produce a senescence‐associated secretory phenotype (SASP) involving the secretion of several chemokines and cytokines.^25^, ^26^ These secretomes alter tumor microenvironments, enhancing epithelial‐to‐mesenchymal transition (EMT) and angiogenesis in vitro and in vivo.^6^, ^27^, ^28^ Furthermore, the soluble factors secreted by senescent cells induce apoptotic resistance to paclitaxel in ovarian cancer,^29^ and in lung cancer, senescence is strongly associated with cancer metastasis.^30^ Molecular mechanisms regulating this cellular aging process are highlighted as potential targets for cancer therapy. Our results demonstrated for the first time the essential role of CAMSAP3 in cellular senescence‐associated phenotypes in human lung carcinoma cells. *CAMSAP3* deletion decreased the cell growth rate, potentiated senescence‐like phenotypes, and upregulated SASP expression (Figures 1 and 2). Consistent with a previous study, we reported that cell proliferation in an attachment condition was decreased in *CAMSAP3* knockout lung cancer cells.^19^ Accordingly, we investigated in detail the underlying mechanism and found that, in the absence of CAMSAP3, ERK phosphorylation is strongly suppressed, consequently inducing cell cycle arrest and mediating senescence‐like phenotypes. Although CAMSAP3 regulated ERK phosphorylation independent of their direct interaction, it maintained the vimentin‐p‐ERK complex that promoted ERK activation.

Morphological alteration, including a flattened cell body and enlarged cell size, is a fundamental senescent phenotype.^5^ Senescent cells actively increase the vacuolar volume, number of lysosomes, and nuclear size, which are related to the enlarged morphology.^26^ These senescence‐associated cell phenotypes are mediated by cytoskeletal rearrangement, particularly in microtubules and actin filaments.^31^ In our study, *CAMSAP3*‐deficient cells exhibited a flattened morphology and increased sizes compared with control cells (Figure 2A). Because CAMSAP3, a microtubule minus‐end binding protein, is required to modulate microtubule organization and cell morphology,^17^, ^19^ in the absence of CAMSAP3, tubulin acetylation is strongly elevated in NSCLC cells. CAMSAP3 might, at least in part, participate in microtubule dynamics, causing morphological changes in senescent cells. Additionally, senescence is an irreversible cell cycle arrest, particularly in the G1 phase, in response to DNA damage induced by telomere erosion and/or oncogene activation.^25^, ^26^ Several genes and proteins regulating the G1 phase, including cyclin D1, CDK4, and p16, contribute to the senescence process.^32^ The irreversible G1 phase cell cycle arrest is initiated by inactivation of cyclin D1‐Cdk4/6 complexes that positively regulate the G1/S phase transition. Furthermore, p16, a cyclin D1‐Cdk4/6 inhibitor, is involved in senescence activity by inhibiting pRB phosphorylation, resulting in the blockade of the E2F transcription factor family.^32^, ^33^ Interestingly, our results demonstrated only the cyclin D1 gene and protein expression levels were consistently decreased in response to *CAMSAP3* knockout (Figure 2C, D). Multiple factors influence the mRNA stability of CDK4 and p16 before the translation of functional proteins such as microRNAs (miRNAs)^34^, ^35^ and RNA decay‐promoting proteins.^36^ MiRNAs have emerged as regulators during the transcription process of cell cycle and senescence markers that directly bind to specific sites in the 3’ untranslated region (3'UTR) of the target mRNA, leading to mRNA degradation or protein translational suppression.^36^ MiR‐124 targets CDK4 mRNA and inhibits protein expression in breast cancer cells.^34^ Furthermore, p16 mRNA is a specific target of the RNA binding protein AUF1, which participates in the RNA degradation machinery.^35^ We, therefore, performed western blotting to confirm their protein levels represented as effector proteins. Our data demonstrated that only cyclin D1 mRNA and protein were concomitantly affected in the absence of CAMSAP3 (Figure 2C, D), indicating that CAMSAP3 regulates cellular senescence‐associated phenotypes by modulating cyclin D1 expression in NSCLC cells.

**FIGURE 7:**
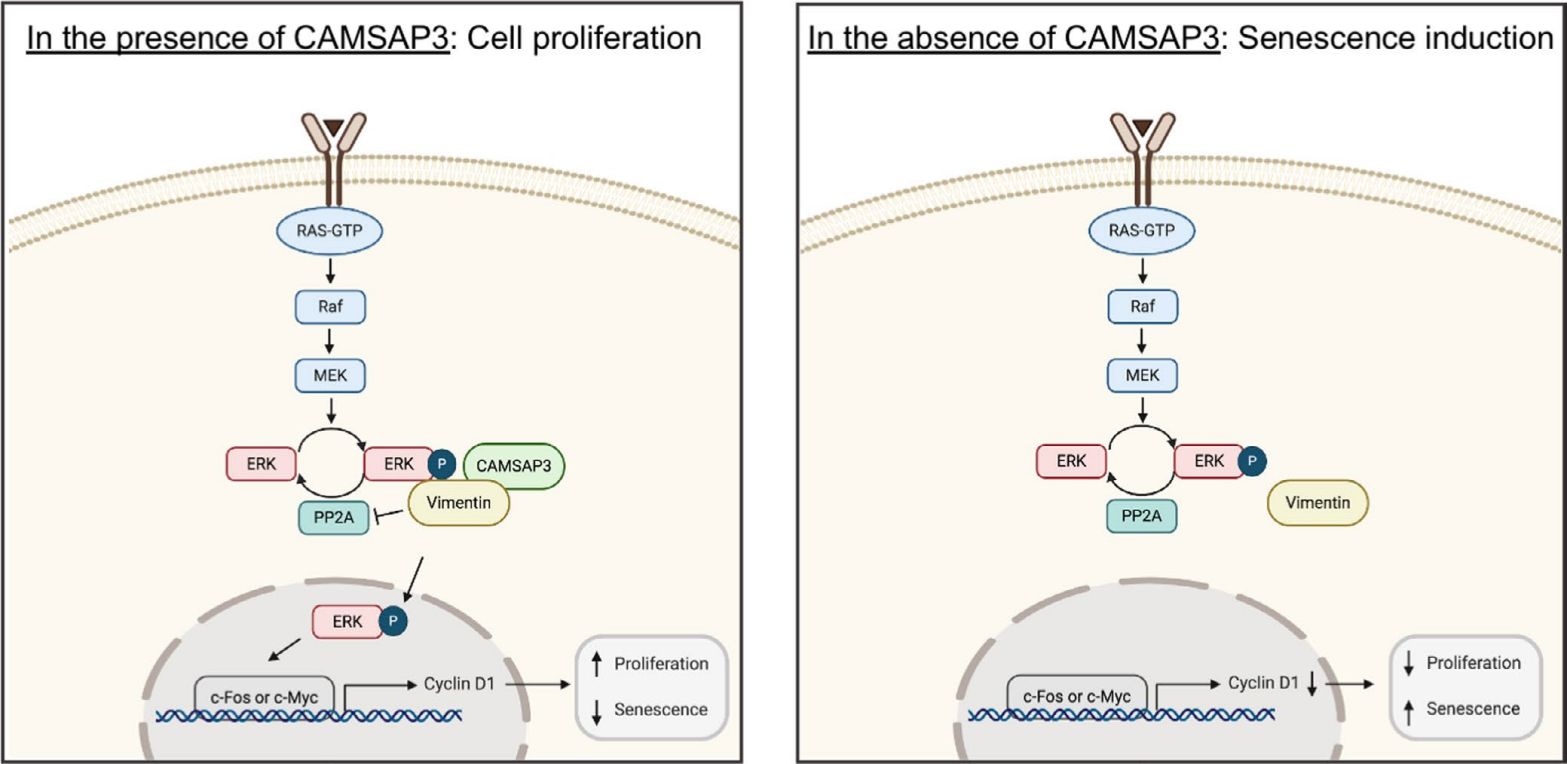
Model for CAMSAP3 regulating NSCLC senescence‐associated phenotypes. In the presence of CAMSAP3 (left), CAMSAP3 binds to vimentin and maintains vimentin‐p‐ERK interaction, consequently stabilizing ERK activation. An active ERK promotes cyclin D1 expression and induces normal cell proliferation. In the absence of CAMSAP3 (right), the interaction of vimentin and p‐ERK is reduced. Thus, senescence‐associated phenotypes are activated

Emerging evidence has demonstrated that ERK is a key messenger of cellular signaling that influences several critical functions, including cell growth, cell survival, and migration.^10^, ^11^ ERK plays dual biological roles in the signal transduction of cell proliferation‐related processes depending on the coordinated pathway and cell type.^15^ ERK‐promoted cell proliferation involves various phosphorylation cascades that regulate several transcription factors of growth‐related genes, such as Elk1, c‐Myc, and c‐Fos.^10^ Many studies on cellular senescence regulation have demonstrated that ERK induces cellular senescence through multiple mechanisms, including upregulating the expression of p21 tumor suppressor protein^11^ and histone H3K27me3 demethylase.^37^ By contrast, suppression of the ERK pathway has been reported to induce senescence phenotypes by decreasing cytoplasmic p16 accumulation in senescent lung fibroblasts,^38^ upregulating p27 expression in A172 glioblastoma cells,^39^ and increasing ROS levels induced by mitochondrial damage in A549 NSCLC cells.^40^ Similar to our finding, p‐ERK appeared to be reduced in the presence of cellular senescence‐like characteristics in *CAMSAP3* knockout lung cancer cells (Figure 3). Furthermore, the introduction of full‐length ERK, but not its dominant mutation, into these cells rescued the above features. Because ERK is a crucial regulator of cyclin D1 and inactivation of ERK downregulates cyclin D1 expression,^41^ *CAMSAP3* loss‐induced cellular senescence‐associated phenotypes is attributed to attenuation of the p‐ERK activity/cyclin D1 axis (Figure 7).

Vimentin, an intermediate filament, plays a crucial role in cellular senescence, not only facilitating morphological changes but also regulating signaling pathways related to this aging process.^42^ The active status of ERK is mainly governed by the balance between ERK phosphorylation and dephosphorylation,^15^ in which vimentin has been reported to be a key determinant. Vimentin enhances ERK phosphorylation by preventing ERK dephosphorylation by the alkaline phosphatase enzyme, contributing to overall ERK activity.^24^ Recently, vimentin was shown to be transported bidirectionally along the microtubule cytoskeleton,^43^ and CAMSAP family proteins regulate the trafficking of cargo through microtubule dynamics.^16^ CAMSAP3 might probably interact with vimentin in this event. Proteomic analysis also indicated an interplay among CAMSAP3, ERK, and vimentin (Figure 5), suggesting that vimentin is an intermediate scaffold for CAMSAP3‐regulated ERK activity. In the absence of CAMSAP3, vimentin could not bind to and properly stabilize ERK phosphorylation, making p‐ERK susceptible to dephosphorylation and resulting in decreased active ERK levels.

Furthermore, our in vivo xenograft experiment demonstrated that the tumor incidence rate at an early phase of tumor implantation was significantly lower in the H460/C3ko groups than in the H460/Ctrl group, although the tumor volume was not different at the end of the experiment (Figure 6). Because *CAMSAP3* knockout cells exhibited looser cell‐cell contacts and an enlarged cell size, the tumor dimension measurements might be affected by these cellular characteristics, as similarly observed in the colony formation assay that cell‐cell interactions also influence this parameter (Figure 1E). Therefore, we used the tumor weight and cell number/field as the readout parameters in an in vivo xenograft model (Figure 6E, F). We also examined necrosis, cell proliferation, senescence‐like phenotypes and SASP expression in these tumor tissues. Once primary cancers are overgrown, a subpopulation located at the center of the tumor tissue can become necrotic because of a lack of growth‐promoting support.^44^ Senescent cells are susceptible to stress‐induced necrotic cell death and further develop inflammation through several mechanisms.^45^ Severe stress, such as prolonged xenograft generation, might contribute to the necrotic cell death observed at the center of the tumor nodule in the *CAMSAP3* knockout group, concomitant with a lower ki67 level and an increase in SA‐β‐gal staining and SASP, indicating the regulatory effect of CAMSAP3 on senescence‐associated phenotypes in an in vivo xenograft.

Interestingly, gene profiling data revealed that CAMSAP3 expression tends to increase in the early stage and decline in the advanced stage of lung cancer.^20^ Our previous study uncovered that *CAMSAP3* knockout enhanced migration ability in lung cancer cells.^19^ In the present study, *CAMSAP3* depletion mediated cellular senescence‐associated phenotypes. CAMSAP3 is differentially expressed during different cancer stages. We hypothesize that, at an early stage, CAMSAP3 expression is upregulated in cancer and promotes tumor initiation. Thus, the loss of CAMSAP3‐delaying tumor growth might be due to, at least in part of, the senescence‐associated phenotypes, and CAMSAP3 levels become gradually downregulated at the advanced stage, facilitating cancer metastasis. However, the functions of CAMSAP3 in cancer biology are largely unexplored. Furthermore, the clinical investigation of CAMSAP3 is necessary to improve its potential application.

## CONFLICT OF INTEREST

The authors declare no conflicts of interest.

## ETHICS APPROVAL

The animal study was approved by the Institutional Animal Care and Use Committee of the Blind for review (CU‐AUP 19‐33‐003).

## Supporting information

Fig S1

Fig S2

Fig S3

Fig S4

Fig S5

Method S1

## Data Availability

Data are available on request from the authors.
