# Supplementary figures and images for: CAMSAP3 depletion induces lung cancer cell senescence‐associated phenotypes through extracellular signal‐regulated kinase inactivation
Source: Cancer Med. 2021 Nov 1;10(24):8961–75. doi: 10.1002/cam4.4380 (PMC8683528; doi:10.1002/cam4.4380)

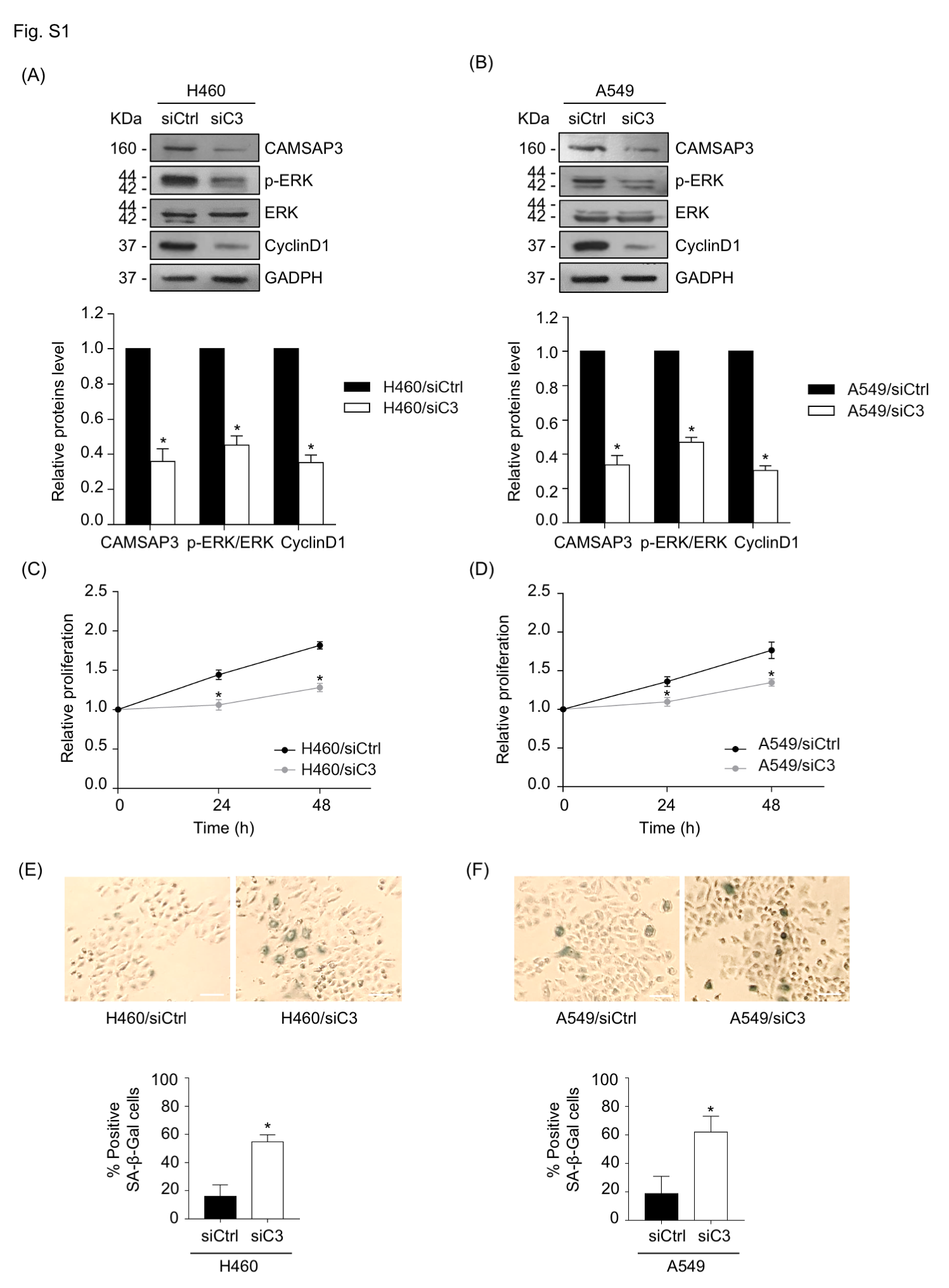

Supplement: Supplementary file 1 — Fig S1 [file CAM4-10-8961-s006.tiff]

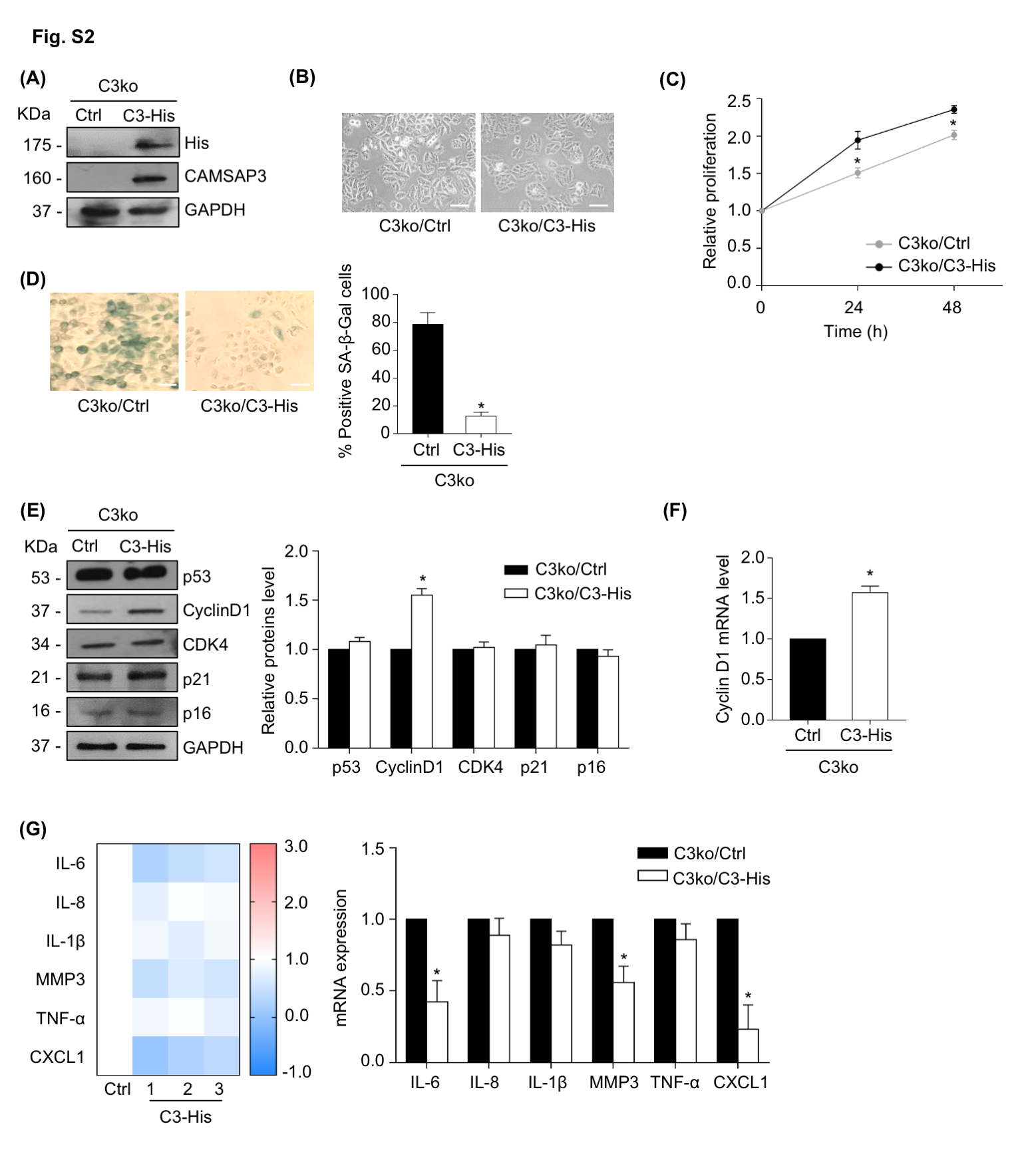

Supplement: Supplementary file 2 — Fig S2 [file CAM4-10-8961-s004.tiff]

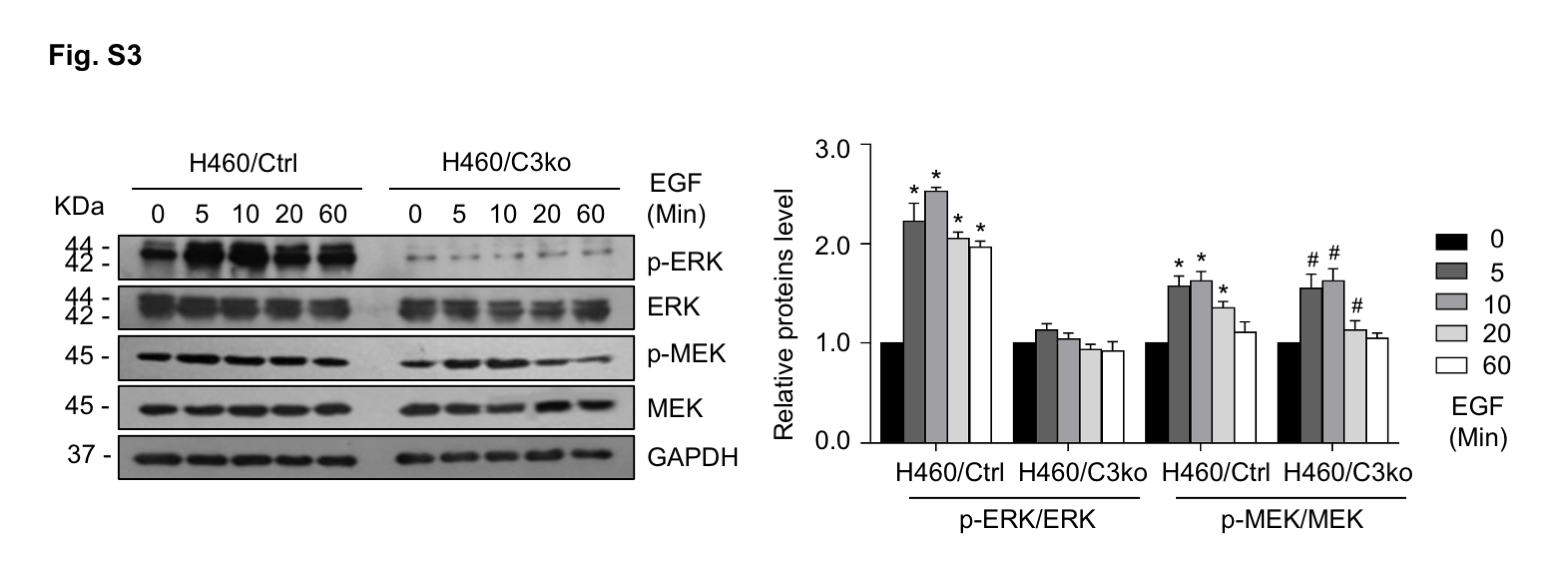

Supplement: Supplementary file 3 — Fig S3 [file CAM4-10-8961-s002.tiff]

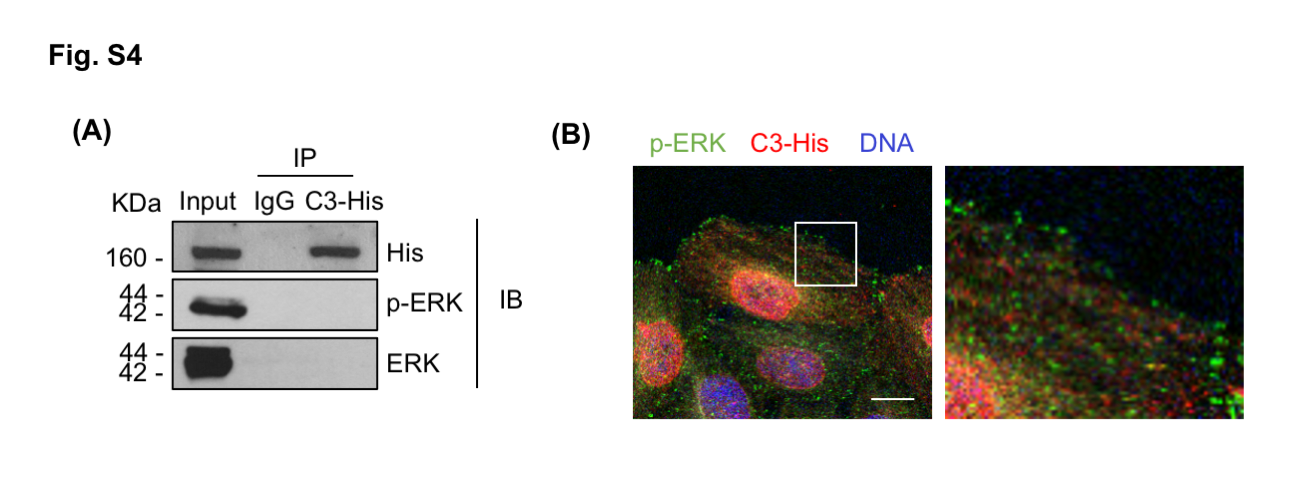

Supplement: Supplementary file 4 — Fig S4 [file CAM4-10-8961-s003.tiff]

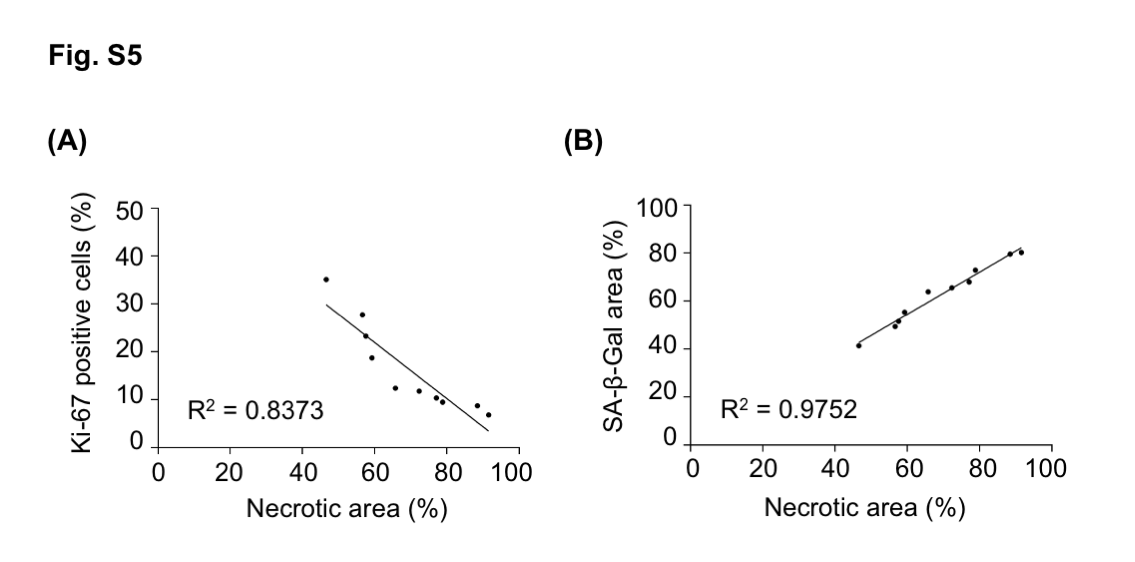

Supplement: Supplementary file 5 — Fig S5 [file CAM4-10-8961-s001.tiff]
