# Supplementary material for: CAMSAP3 depletion induces lung cancer cell senescence‐associated phenotypes through extracellular signal‐regulated kinase inactivation
Source: Cancer Med. 2021 Nov 1;10(24):8961–75. doi: 10.1002/cam4.4380 (PMC8683528; doi:10.1002/cam4.4380)
Supplement: Supplementary file 6 — Method S1 [file CAM4-10-8961-s005.docx]

**Supportive Information for**

**CAMSAP3 depletion induces lung cancer cell senescence-associated phenotypes through extracellular signal-regulated kinase inactivation**

Onsurang Wattanathamsan^1,2^, Paninee Chetprayoon^3^, Naphat Chantaravisoot^4,5^, Piriya Wongkongkathep^5^, Pithi Chanvorachote^6,7^, Varisa Pongrakhananon^2,6*^

^1^ Inter-department program of Pharmacology, Graduate School, Chulalongkorn University, Bangkok, 10330, Thailand

^2^ Preclinical Toxicity and Efficacy Assessment of Medicines and Chemicals Research Unit, Chulalongkorn University, Bangkok 10330, Thailand

^3^ Toxicology and Bio Evaluation Service Center, National Science and Technology Development Agency, Pathum Thani 12120, Thailand

^4^ Department of Biochemistry, Faculty of Medicine, Chulalongkorn University, Bangkok 10330, Thailand

^5^ Center of Excellence in Systems Biology, Faculty of Medicine, Chulalongkorn University, Bangkok 10330, Thailand

^6^ Department of Pharmacology and Physiology, Faculty of Pharmaceutical Sciences, Chulalongkorn University, Bangkok 10330, Thailand

^7^ Cell-based Drug and Health Product Development Research Unit, Faculty of Pharmaceutical Sciences, Chulalongkorn University, Bangkok 10330, Thailand

Correspondence: Varisa Pongrakhananon, Department of Pharmacology and Physiology, Faculty of Pharmaceutical Sciences, Chulalongkorn University, Bangkok 10330, Thailand

Tel: +662-218-8325, Fax: +662-218-8340, Email: [varisa.p@pharm.chula.ac.th](mailto:varisa.p@pharm.chula.ac.th)

**SUPPLEMENTARY METHODS**

**Plasmid construction and transfection**

To construct mutant ERK1 cDNAs, mutagenesis was conducted using a site-direct mutagenesis kit (‎Thermo Fisher Scientific). Briefly, the ERK1 mutant (T202A/Y204F) was generated by amplification from a human ERK1-wild type plasmid (Addgene #14747) using a mutagenic PCR kit with specific primers. The phosphorylation site at T202 was replaced by alanine, and Y204 by phenylalanine, using the sense primer *5’-TGGCGGAGTTTGTGGCTACG-3’* and antisense primer *5’- TAGTTTCGGGCCTTCATGTT -3’*. The PCR products were subcloned into pEGFP-C1. GFP-ERK1^T202A/Y204F^ plasmid construction was verified by DNA sequencing. The His-tagged CAMSAP3 full-length expressing plasmid was constructed as previously described^1^.

The plasmid was transfected into the cells using Lipofectamine^®^ 2000 (Invitrogen). Briefly, 2 μg of plasmid in optiMEM media was incubated with 4 μl of Lipofectamine^®^ 2000. After 15 min, the plasmid-Lipofectamine mixture was added dropwise into the cell culture followed by incubation at 37 °C for 6 h. To generate stable transfectants, the cells were treated with 400 µg/ml of G418 for at least 7 d, followed by constitutive colony selection using the serial dilution method. Exogenous DNA expression was confirmed by western blotting or immunofluorescence assay.

**RNA extraction and RT-PCR**

RNA was extracted using GENEzol reagent (Geneaid Biotech). One microgram of RNA was reverse transcribed to cDNA using SuperScript^TM^ III Reverse Transcriptase (Invitrogen). The mRNA expression levels of cell cycle and senescence regulatory genes were determined using a Step one plus real-time PCR system and the SensiFAST^TM^ SYBR^®^ NO-ROX Kit (Bioline). The primers are shown in Table S1. Real-time PCR was assessed using a StepOnePlus Real-Time PCR system (Applied Biosystems). The thermocycling conditions were set as follows: 95 °C for 10 min, 95 °C for 30 s (35 cycles) and 60 °C for 30 s. The data were analyzed using the Ct^-ΔΔ^ method^2^.

**Western blot analysis**

Cells were incubated in TMN lysis buffers as previously described^1^. An equal protein amount was separated by SDS-PAGE, followed by transfer to PVDF membranes. The blots were blocked with 5% skim milk in TBS-T buffer (Tris buffer saline with 0.075% Tween-20) and consequently incubated with a specific primary antibody at 4 °C overnight. After that, the blots were incubated with secondary antibody at room temperature for 2 h. Protein expressions were detected by the chemiluminescence system (Merck Millipore). The blots were reprobed with anti-GAPDH to confirm equal loading. The relative protein intensity was measured and normalized to GAPDH using ImageJ software (NIH). The data were analyzed from triplicate experiments.

**Immunofluorescence assay**

Cells were fixed with cold methanol for 5 min at -20 °C. After that, the cells were permeabilized in 0.1% Triton X-100 in PBS, blocked with 3% BSA, incubated with primary antibodies at 4 °C overnight and secondary antibody at room temperature for 2 h in the dark^1^. Images were observed using a confocal microscope (Leica TCS SP8) with a 100× oil immersion objective lens. The fluorescence intensity obtained from at least 20 cells was analyzed using ImageJ software (NIH), and colocalization of signals was analyzed using the JACoP plugin^3^.

**Immunoprecipitation assay**

Cells were dissolved in a lysis buffer as previously described^1^. The supernatant was collected by centrifugation at 20,000× g and 4 °C for 20 min, and then a pre-cleared step was performed using Protein G-conjugated Sepharose beads (GE Healthcare) at 4 °C for 1 h. The supernatant was then separated by centrifugation and incubated with a specific antibody or IgG as the control at 4 °C overnight. The protein complexes were pulled down by incubating with Protein G-conjugated Sepharose beads at 4 °C for 1 h. After washing, the precipitate was heated in a sample buffer at 95 °C for 5 min and subjected to western blot analysis as described above. The representative blots are shown from triplicate independent experiments.

**Immunoprecipitation and mass spectrometry assay (IP-MS)**

Co-immunoprecipitation was performed by pulling down ERK and his-tagged CAMSAP3 using anti-ERK and anti-his, respectively. The ERK and CAMSAP3 his-tagged preparations were fractionated by SDS-PAGE, and gels were stained using Coomassie blue to detect and excise specific bands for mass spectrometry analysis. Proteins were reduced with 10 mM dithiothreitol and alkylated with 50 mM iodoacetamide. In-gel trypsin digestion was performed at 37 °C, and proteins were identified by liquid chromatography-tandem mass spectroscopy (LC-MS/MS) as previously described^4^. The data were analyzed using Proteome Discoverer 2.1 (Thermo Fisher Scientific) and searched against a uniport human protein database (UP000005640, 20370 entries) to identify proteins entrapped in gel slices. At least three unique peptides were identified, and pathway analysis was evaluated using the Reactome pathway database^5^.

**Immunohistochemistry**

After deparaffinization with xylene and 95%-100% ethanol, 0.1 M citrate buffer (pH 6.0) was added to the sections, followed by microwave heating for antigen retrieval. Each 4-μm-thick section was incubated with 100-300 µl of 3% H_2_O_2_ for 15 min and blocked with 5% non-fat dry milk in TBS-T for 30 min at room temperature. The sections were incubated with primary antibodies against ki-67 overnight at 4˚C, washed twice with PBS-T, and incubated with HRP-conjugated DAB (Wako) for 2 h at room temperature. The sections were then washed with PBS-T and dehydrated, and the mounted slides were observed under a light microscope. The total areas of the tumor slices and necrotic areas from 10 samples were determined using ImageJ software (NIH). Ki-67-positive cells and SA-β-gal areas were analyzed by ImageJ software and calculated as percentages.

**
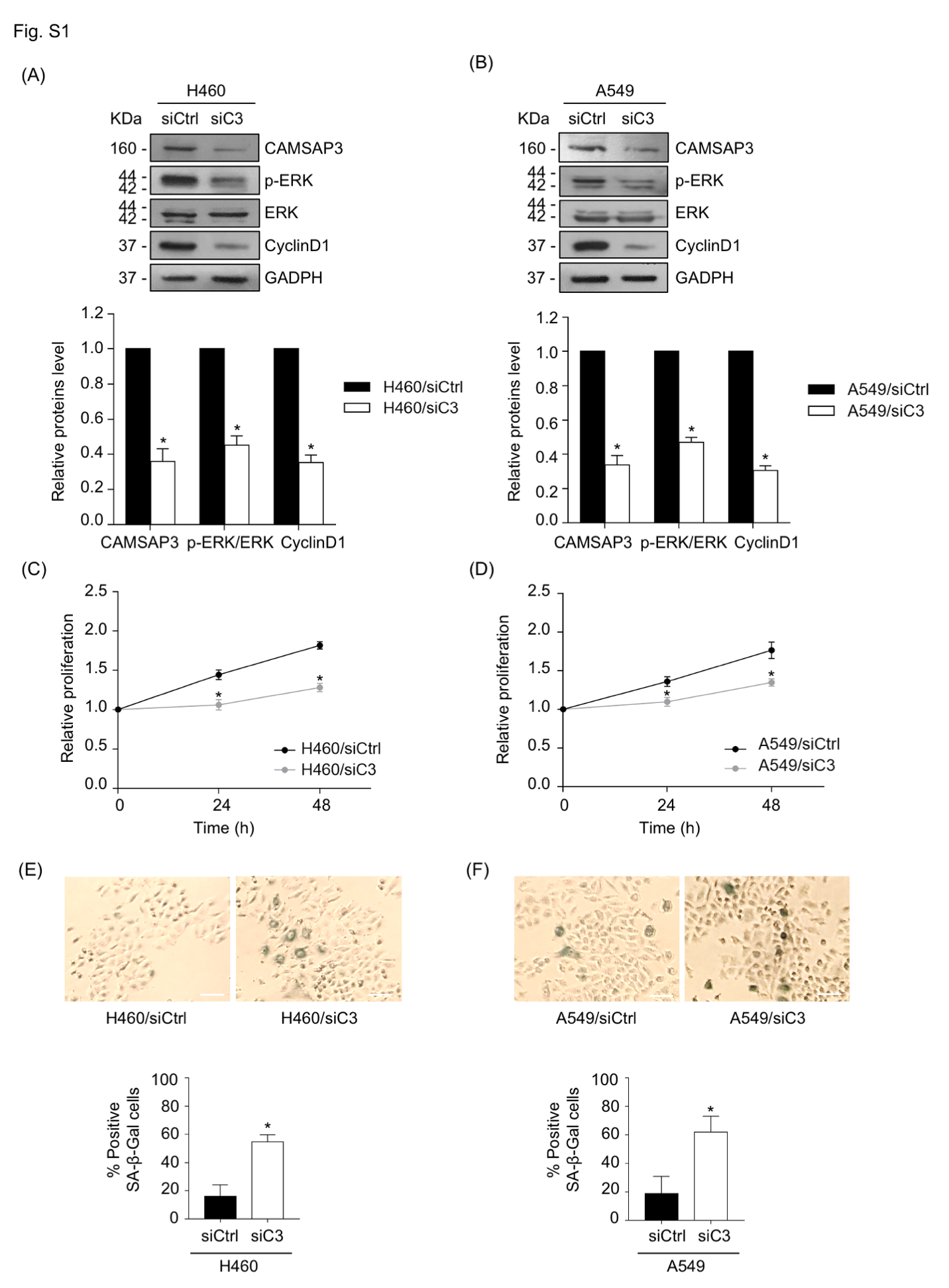
**

**Figure S1** CAMSAP3 Knockdown mediates H460 and A549 cell senescence-associated phenotypes through p-ERK/cyclin D1. Cells were transfected with siRNA target at CAMSAP3 or control, after transfection for 72 h, biochemical analysis was performed. (A) Western blot for CAMSAP3, p-ERK, ERK and cyclin D1 in CAMSAP3 knockdown H460 (H460/siC3) and its control (H460/Ctrl) cells. The intensity was normalized to the intensity of GAPDH. Graph represented mean ± SEM. Student’s *t*-test, **p* < 0.05 vs H460/siCtrl cells. (*n=3*). (B) Western blot for CAMSAP3, p-ERK, ERK and cyclin D1 in CAMSAP3 knockdown A549 (A549/siC3) and its control (A549/Ctrl) cells. The intensity was normalized to the intensity of GAPDH. Graph represented mean ± SEM. Student’s *t*-test, **p* < 0.05 vs A549/siCtrl cells. (*n=3*). (C) H460 and (D) A549 cell proliferation was examined by MTT assay and represented as a mean ± SEM of the relative value. Student’s *t*-test, **p* < 0.05 vs control cells. (*n=3*). Representative phase contrast images of SA-β-Gal-stained. (E) H460 and (F) A549 cells (positive blue stained cells). Graph represented mean ± SEM of positive SA-β-Gal cells. Student’s *t*-test, **p* < 0.05 vs siCtrl cells. (*n=3*) Scale bars: 10 µm.


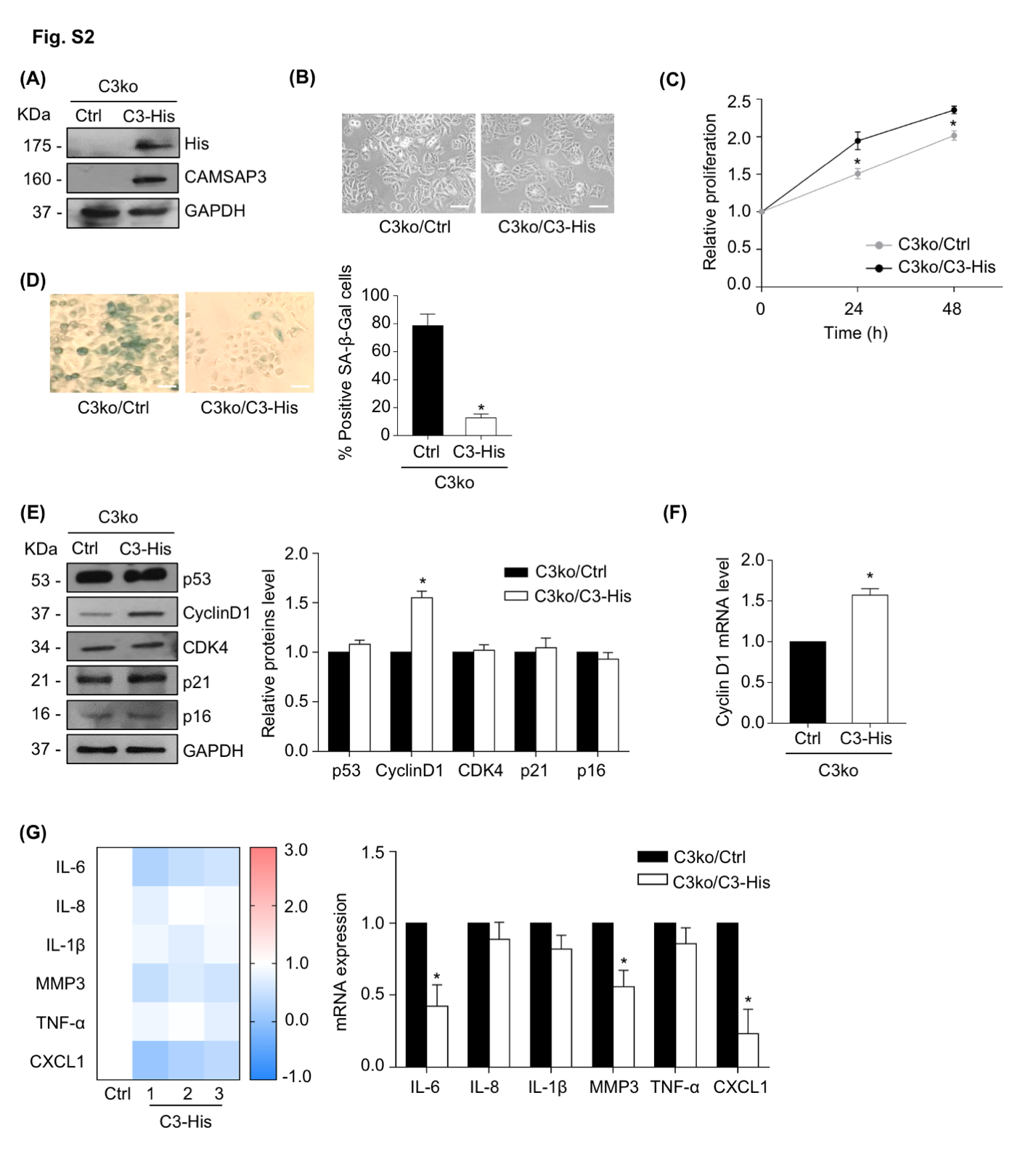


**Figure S2** Overexpression CAMSAP3-wild type (C3-His) inhibits senescence-associated phenotypes in *CAMSAP3* knockout cells. (A) Western blot of exogenous His-tagged CAMSAP3 in *CAMSAP3* knockout (C3ko/C3-His) and their control (C3ko/Ctrl) cells. (B) Phase-contrast images of C3ko/Ctrl and C3ko/C3-His cells. Scale bars: 10 µm. (C) Cell proliferation of C3ko/Ctrl and C3ko/C3-His cells were examined by MTT assay and represented as a mean ± SEM of the relative value. Student’s *t*-test, **p* < 0.05 vs C3ko/Ctrl cells (*n=3*). (D) Representative phase contrast images of SA-β-Gal-stained cells (positive blue stained cells). Graph represented mean ± SEM of positive SA-β-Gal cells. Student’s *t*-test, **p* < 0.05 vs C3ko/Ctrl cells. (*n=3*) Scale bars: 10 µm. (E) Western blot for cell cycle and senescence regulatory proteins in C3ko/Ctrl and C3ko/C3-His cells. The intensity was normalized to the intensity of GAPDH. Graph represented mean ± SEM. Student’s *t*-test, **p* < 0.05 vs C3ko/Ctrl cells (*n=3*). (F) The mRNA expression levels of cyclin D1 was measured by quantitative RT-PCR. The data are presented as mean ± SEM. Student’s *t*-test, **p* < 0.05 vs C3ko/Ctrl cells (*n=3*). (G) The mRNA expression levels of SASP were measured by quantitative RT-PCR. The heatmap represented the relative mRNA level compared with those of the C3ko/Ctrl cells. The data are presented as mean ± SEM. Student’s *t*-test, **p* < 0.05 vs H460/Ctrl cells (*n=3*).


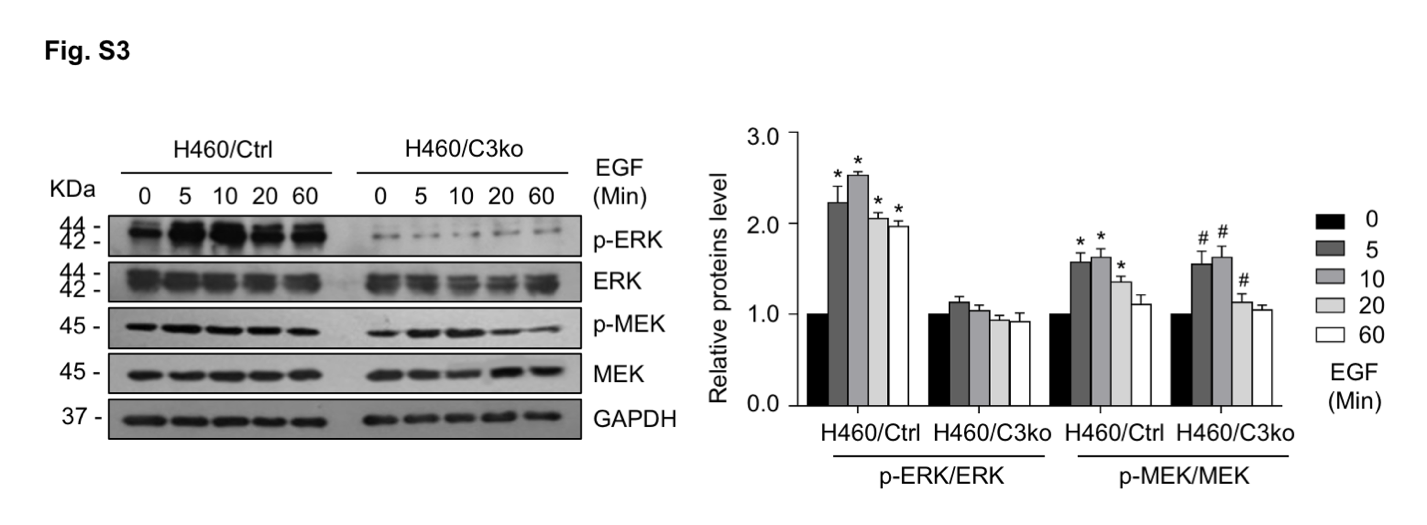


**Figure S3** CAMSAP3 has no effect on an upstream of MAP kinase pathway. Western blot for p-MEK1/2, MEK, p-ERK and ERK in H460/Ctrl cells and H460/C3ko cells after treatment with 100 ng/mL epidermal growth factor (EGF) for the indicated time points. The intensity was normalized to that of GAPDH. Data was presented as means ± SEM. *P*-values were calculated using a Student’s *t*-test **p* < 0.05 vs H460/Ctrl cells at time 0 min; #*p* < 0.05 vs C3ko/Ctrl cells at time 0 min (*n=3*).


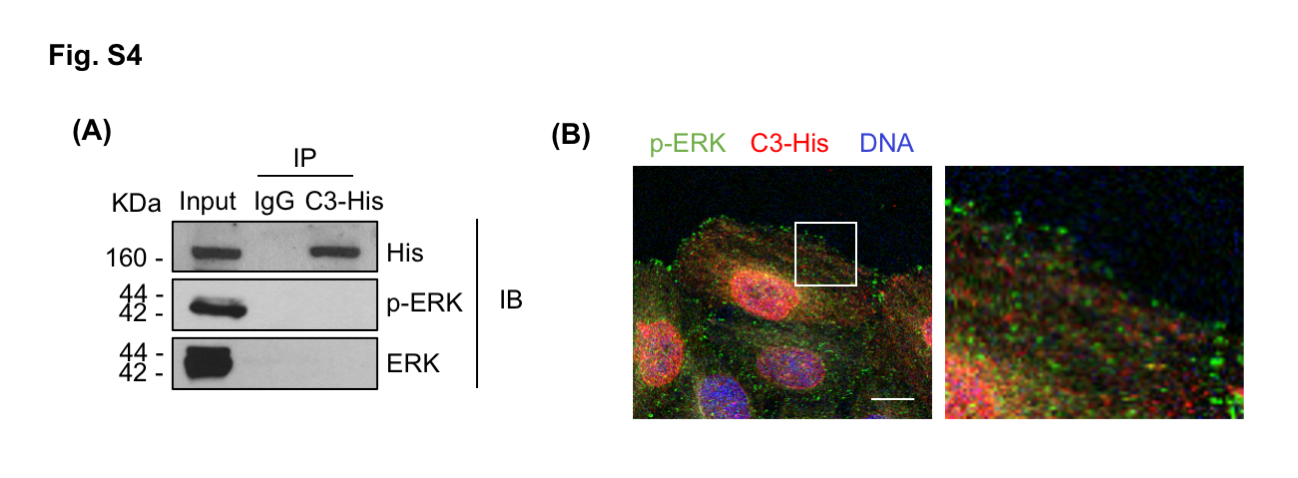


**Figure S4** There is no interaction among CAMSAP3 to ERK and p-ERK. (A) H460 cells were transfected with His-tagged CAMSAP3 and the lysates were immunoprecipitated with anti-His antibody or IgG as negative control. The precipitates were then subjected to immunoblotting for His, ERK and p-ERK. (B) Immunostaining for p-ERK (green), His-tagged CAMSAP3 (C3-His, red) and DNA (DAPI, blue) in CAMSAP3 overexpressing His-tagged H460 cells. Box was enlarged on the right. Scale bars: 10 µm.


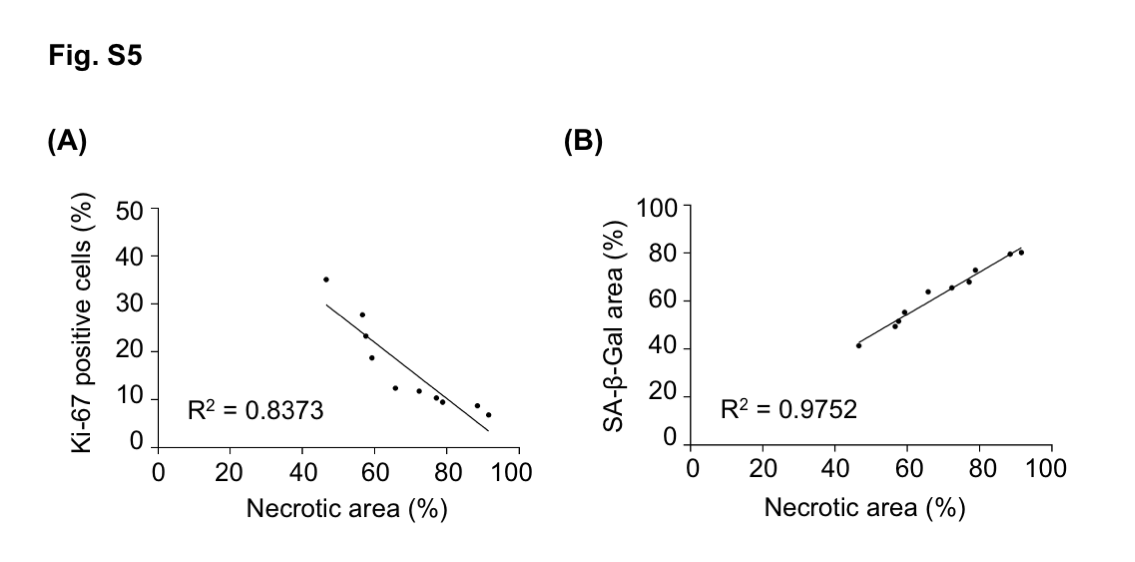


**Figure S5** The correlation of the necrosis-ki67 and necrosis-SA-β-gal level in the H460/C3ko group. (A) The plot represents a correlation analysis performed between the percentage of necrosis area and ki67 expression in each H460/C3ko group. (B) The plot represents a correlation analysis performed between the percentage of necrosis area and SA-β-Gal expression in each H460/C3ko group.

**Table S1** List of primer sequences used for RT-PCR analysis in this study

| Genes | Primer sequences (5'-3') | |
| --- | --- | --- |
|  | F’: Forward primer | R’: reverse primer |
| cyclin D1 | F’:GATGCCAACCTCCTCAACGA | R’:TCGCAGACCTCCAGCATCCA |
| CDK4 | F’:CTGGTGTTTGAGCATGTAGACC | R’:AAACTGGCGCATCAGATCCTT |
| CDK6 | F’:TGGAGACCTTCGAGCACC | R’:CACTCCAGGCTCTGGAACTT |
| cyclin E | F’:ATCAGCACTTTCTTGAGCAACA | R’:TTGTGCCAAGTAAAAGGTCTCC |
| CDK2 | F’:CCAGGAGTTACTTCTATGCCTGA | R’:TTCATCCAGGGGAGGTACAAC |
| p16 | F’:GCCCAACGCACCGAATAG | R’:ACGGGTCGGGTGAGAGTG |
| p21 | F’:CGATGGAACTTCGACTTTGTCA | R’:GCACAAGGGTACAAGACAGTG |
| p27 | F’:TCTGAGGACACGCATTTGGT | R’:ACAGAACCGGCATTTGGGG |
| p53 | F’:TAACAGTTCCTGCATGGGCGGC | R’:AGGACAGGCACAAACACGCACC |
| IL-6 | F’:TACCCCCAGGAGAAGATTCC | R’:TTTTCTGCCAGTGCCTCTTT |
| IL-8 | F’:GTGCAGTTTTGCCAAGGAGT | R’:CTCTGCACCCAGTTTTCCTT |
| IL-1β | F’:CCACAGACCTTCCAGGAGAATG | R’:GTGCAGTTCAGTGATCGTACAGG |
| CXCL1 | F’:AGGGAATTCACCCCAAGAAC | R’:TGGATTTGTCACTGTTCAGCA |
| TNF-α | F’:CAGCCTCTTCTCCTTCCTGAT | R’:CTCAGCTTGAGGGTTTGCTAC |
| MMP3 | F’:ATGGACAAAGGATACAACAGGGA | R’:GTGAGTGAGTGATAGAGTGGG |
| LMNB1 | F’: AGCGGAAGAGGGTTGATGTG | R’: CCAGCCTCCCATTGGTTGAT |

**Table S2** List of primary and secondary antibodies, their company, catalog number, host species and working dilution

| Antibody | Company | Catalog number | Host species | Working Concentration | |
| --- | --- | --- | --- | --- | --- |
|  |  |  |  | WB | IF |
| CAMSAP3 |  |  | Rabbit | 1:500 | 1:200 |
| p-ERK | Cell Signaling Technology | #4376 | Rabbit | 1:1000 | - |
| p-ERK | Cell Signaling Technology | #9101 | Rabbit | - | 1:1000 |
| ERK | Cell Signaling Technology | #4695 | Rabbit | 1:1000 | - |
| p53 | Cell Signaling Technology | #2524 | Mouse | 1:1000 | - |
| GAPDH | Cell Signaling Technology | #97166 | Mouse | 1:1000 | - |
| Tubulin | Sigma | # T6199 | Mouse | 1:5000 | 1:1000 |
| Vimentin | Santa Cruz Biotechnology | #sc-6260 | Mouse | 1:1000 | - |
| p16 | Santa Cruz Biotechnology | #sc-377412 | Mouse | 1:1000 | - |
| p21 | Santa Cruz Biotechnology | #sc-271532 | Mouse | 1:1000 | - |
| cyclin D1 | Santa Cruz Biotechnology | # sc-8396 | Mouse | 1:1000 | - |
| CDK4 | Santa Cruz Biotechnology | # sc-23896 | Mouse | 1:1000 | - |
| His | MBL | # D291-3 | Mouse | 1:1000 | 1:500 |
| α-tubulin | EMD Millipore | # MAB1864 | Rat | 1:1000 | - |
| GFP | Abcam | #13970 | Chicken | 1:1000 | - |
| Alexa Fluor 488 | Invitrogen | #A11034 | Rabbit | - | 1:1000 |
| Alexa Fluor 568 | Invitrogen | #A11032 | Mouse | - | 1:1000 |
| Alexa Fluor 647 | Invitrogen | #A21247 | Rat | - | 1:1000 |
| HRP-conjugated anti-rabbit | Cell Signaling Technology | #7074 | Rabbit | 1:1000 | - |
| HRP-conjugated anti-Mouse | Cell Signaling Technology | #7076 | Mouse | 1:1000 | - |

**References**

1. Pongrakhananon V, Wattanathamsan O, Takeichi M, Chetprayoon P, Chanvorachote P. Loss of CAMSAP3 promotes EMT via the modification of microtubule–Akt machinery. *J Cell Sci.* 2018;131:jcs216168.

2. Livak KJ, Schmittgen TD. Analysis of relative gene expression data using real-time quantitative PCR and the 2− ΔΔCT method. *Methods.* 2001;25:402–408.

3. Bolte S, Cordelières FP. A guided tour into subcellular colocalization analysis in light microscopy. *J Microsc.* 2006;224:213–232.

4. Chantaravisoot N, Wongkongkathep P, Loo JA, Mischel PS, Tamanoi F. Significance of filamin A in mTORC2 function in glioblastoma. *Mol Cancer.* 2015;14:1–14.

5. Fabregat A, Sidiropoulos K, Viteri G, Marin-Garcia P, Ping P, Stein L, et al*.* Reactome diagram viewer: data structures and strategies to boost performance. *Bioinformatics.* 2018;34:1208–1214.
